# lnc-ALX1-2:10 is a novel regulator that enhances proliferation, migration and invasion in prostate cancer cells

**DOI:** 10.1038/s41598-026-42299-0

**Published:** 2026-03-03

**Authors:** Xinjun Wang, Qian Zong, Yue Bu, Bin Zhou, Xuqiang Wang, Zhangqun Li, Guangcheng Luo

**Affiliations:** 1 Department of Urology, Zhongshan Hospital Xiamen University, School of Medicine，Xiamen University, Xiamen, 361004 China; 2 The Graduate School of Fujian Medical University, Fuzhou, 350122 China; 3 Zhongshan Hospital Xiamen University, No.209 Hubin South Road, Xiamen, 361004 China

**Keywords:** Lnc-ALX1-2:10, Migration, Proliferation, Prostate cancer, Transcriptome sequencing, Cancer, Cell biology, Molecular biology, Oncology

## Abstract

Although long noncoding RNAs (lncRNAs) have been implicated in the progression of prostate cancer (PCa), the functional roles of many of these molecules, especially in metastasis, remain poorly understood. To identify novel lncRNAs linked to PCa malignant phenotypes, we analyzed the differences in lncRNA expression between the highly metastatic PCa cell line (PC-3 M-1E8) and the poorly metastatic PCa cell line (PC-3 M-2B4) using transcriptome sequencing. This differential expression was confirmed by RT-qPCR, which showed that the lnc-ALX1-2 gene cluster (including lnc-ALX1-2:5, lnc-ALX1-2:7, lnc-ALX1-2:10) was significantly upregulated in PC-3 M-1E8 cells. Functional studies demonstrated that knockdown of the lnc-ALX1-2 gene cluster suppressed proliferation, migration, and invasion in PC-3 M-1E8 cells, with lnc-ALX1-2:10 showing the most prominent effect. Transcriptome analysis further revealed that lnc-ALX1-2:10 knockdown altered the expression of 194 genes related to both proliferation and migration. RT-qPCR and Western blot validated that lnc-ALX1-2:10 knockdown downregulated pro-tumorigenic factors (CCNE1, PDGFRA, ANGPT4) and EMT markers (N-cadherin, Snail, Vimentin), while upregulating ITGAL and E-cadherin. In vivo, lnc-ALX1-2:10 knockdown obviously decreased tumor volume but had no effect on mouse body weight, and molecular analysis of xenograft tumors confirmed consistent expression changes of key proteins as in vitro. Collectively, our findings identify lnc-ALX1-2:10 as a novel lncRNA that promotes aggressive phenotypes in PCa, highlighting its potential as a therapeutic target for metastatic disease.

**Supplementary Information:**

The online version contains supplementary material available at 10.1038/s41598-026-42299-0.

## Introduction

Prostate cancer (PCa) is the second most frequent cancer in American and European males, and its incidence rate has also increased in other countries over recent years, resulting in the deaths of tens of thousands of males every year globally^1,2^. With the increasing development of the understanding and treatment of PCa, the prognosis for patients with PCa has been greatly improved^3,4^. However, there remain many deficiencies in the research and treatment of PCa. Timely diagnosis, reliable assessment of cancer metastasis, and effective molecular therapeutic targets could provide new insights and directions in PCa diagnosis and treatment.

Long noncoding RNAs (lncRNAs) are a class of endogenous RNAs of more than 200 bp in length that exert biological functions by encoding polypeptides or regulating other RNAs and proteins^5,6^. LncRNAs have a variety of important functions and dysregulated expression or dysfunction of lncRNAs has been related to the occurrence and development of many diseases, including cancer, neurological diseases, and cardiovascular diseases^7–9^. Previous studies have shown that many lncRNAs could be used as potential biomarkers or therapeutic targets in PCa^10^. For example, lncRNA HANR promotes aerobic glycolysis in PCa by stabilizing TPI1^11^, whereas MIR22HG suppresses the development and progression of PCa by targeting miR-4428 and upregulating the expression of PCDH9^12^. However, the understanding of the alterations and functions of lncRNAs in PCa remains limited, especially with respect to the relationship between lncRNAs and the migration and proliferation of PCa cells.

Notably, recent studies have identified a lncRNA, lnc-ALX1-2 (also known as LINC02820), located approximately 16 kb downstream of the ALX1 gene on chromosome 12. This gene gives rise to multiple transcripts, among which lnc-ALX1-2:10 (chr12:85,318,019–85,318,605, GRCh38/hg38) is the focus of the present study. Intriguingly, lnc-ALX1-2/LINC02820 is not a molecule of entirely unknown function. It has been reported to exhibit high nuclear enrichment in invasive bladder cancer cells^13^, and in esophageal squamous cell carcinoma, it promotes tumor growth and metastasis by regulating MYH9 expression or augmenting the TNF signaling pathway to remodel the cytoskeleton^14,15^. Furthermore, its neighboring gene ALX1, a transcription factor, has been shown to drive invasion by inducing epithelial-mesenchymal transition (EMT) in various cancers such as lung and ovarian cancer^16,17^. These findings collectively suggest that ALX1 and lnc-ALX1-2/LINC02820, despite their independent genetic loci, may converge functionally on regulating core malignant phenotypes like cell motility and invasion. However, the expression pattern and functional role of lnc-ALX1-2:10 in PCa remain completely unexplored.

To address this gap and to systematically identify novel functional lncRNAs in PCa metastasis, we performed transcriptome sequencing to analyze the differential expression profiles between a highly metastatic PCa cell line (PC-3 M-1E8) and its poorly metastatic counterpart (PC-3 M-2B4). This approach led us to discover that the lnc-ALX1-2 gene cluster, including the transcript lnc-ALX1-2:10, was significantly upregulated in the highly metastatic cells. Therefore, this study aimed to investigate the specific role of lnc-ALX1-2:10 in the proliferation and migration of PCa cells and to preliminarily explore its underlying molecular mechanisms. Our findings reveal lnc-ALX1-2:10 as a novel regulator that promotes malignant phenotypes in PCa, highlighting its potential as a therapeutic target and a biomarker for metastatic propensity.

## Materials and methods

### Cell culture

PC-3 M-1E8 and PC-3 M-2B4 were purchased from American Type Culture Collection (ATCC, Manassas, VA, USA). These isogenic cell lines, derived from the parental PC-3 M line, are well-established models for studying PCa metastasis, with PC-3 M-1E8 exhibiting significantly higher metastatic potential in vivo and in vitro compared to PC-3 M-2B4, as previously characterized^18,19^. All cells were cultured in Dulbecco’s Modified Eagle Medium (DMEM, Gibco, Grand Island, NY, USA) with 10% fetal bovine serum (FBS, Gibco), 100 U/mL penicillin (Gibco), and 100 µg/mL streptomycin (Gibco) at 37℃ and 5% carbon dioxide.

## RT-qPCR

Total RNA from cells was extracted using TRIzol reagent (Invitrogen, Carlsbad, CA, USA), quantified by Nanodrop, and then reverse-transcribed to cDNA using RevertAid RT Reverse Transcription Kit (Thermo Fisher Scientific, Waltham, MA, USA). Quantitative PCR was performed using ChamQ Universal SYBR qPCR Master Mix (Vazyme, Nanjing, China), and expression levels were normalized relative to that of glyceraldehyde-3-phosphate dehydrogenase (GAPDH). The primer sequences are shown in Table 1.

**Table 1:** Primers sequences in this study.

Gene ID	Sequence (5’ −3’)
LINC00162-F	TGCCTGGACTTTCAAGAGGTAA
LINC00162-R	GCTCTCAGTCAGCAGACACTT
LINC00163:1-F	GACAGGCTCACCAGAACCA
LINC00163:1-R	GGAGGAAGCAGAACCGCA
LINC00163:2-F	TCGGCCTTGGGTTTATCAC
LINC00163:2-R	TGAGGAAGCAGACCGTTGTG
LINC00958-F	AGAGAGGAGGAGAAGCAA
LINC00958-R	TGTGAAGTGCAGGGAGGA
LINC01296-F	AACCCTCATCCATATCCT
LINC01296-R	TGGTTTTCTGGGTTTTGAC
LNC-ALX1-2-F	TCTTCTGTCAACCTGGTGCA
LNC-ALX1-2-R	GTTGATTCAGGAACCCAGGG
LNC-ALX1-2:7-F	GGGGACTGATCGGAGTCAAAC
LNC-ALX1-2:7-R	TCCAGCACCACATTCTCTTCT
LNC-ALX1-2:10-F	TCTTCGCTTTTTATTTCTTTCT
LNC-ALX1-2:10-R	CTTATGTTTGGCAACAACTTTT
H-ANGPT4-F	GCCTATAGCCTGCAGCTCAC
H-ANGPT4-R	AGTACTGGCCGTTGAGGTTG
HGF-F	GCTATCGGGGTAAAGACCTACA
HGF-R	CGTAGCGTACCTCTGGATTGC
TNFSF13B-F	GGGAGCAGTCACGCCTTAC
TNFSF13B-R	GATCGGACAGAGGGGCTTT
CALML6-F	CAGAATCCTGTCAGACAACCAC
CALML6-R	TCGAACATCTCAAAGACTCCCT
RASA4B-F	CAATGGCACATCTGACCCCT
RASA4B-R	AGTGGCTGGTAGTAGCTGGA
DCHS2-F	ATCAGTCCCGTATTACTTCTGGA
DCHS2-R	CCAGTCATGGTCGATGTTTGAA
CCNE1-F	GCCAGCCTTGGGACAATAATG
CCNE1-R	CTTGCACGTTGAGTTTGGGT
WNT7A-F	AGAAGCAAGGCCAGTACCAC
WNT7A-R	GCCTCGTTGTTGTGCAAGTT
PDGFRA-F	TGGCAGTACCCCATGTCTGAA
PDGFRA-F	CCAAGACCGTCACAAAAAGGC
MAPK11-F	AAGCACGAGAACGTCATCGG
MAPK11-R	TCACCAAGTACACTTCGCTGA
C8orf44-SGK3-F	AGCTGCCCAAGTGTAAGCAT
C8orf44-SGK3-R	AGCTGCCCAAGTGTAAGCAT
MYB-F	ATCTCCCGAATCGAACAGATGT
MYB-R	TGCTTGGCAATAACAGACCAAC
ANK1-F	CCAGATGAATGGTTACTCCTCAC
ANK1-R	CAAGGGGATGGCGTCTAGG
CLDN14-F	AGCGGCATGAAGTTTGAGATT
CLDN14-R	CCCGATTGTCTTTGTAGGCAG
ITGAL-F	TGCTTATCATCATCACGGATGG
ITGAL-R	CTCTCCTTGGTCTGAAAATGCT
GAPDH-F	GAGTCAACGGATTTGGTCGT
GAPDH-R	GACAAGCTTCCCGTTCTCAG

## Western blotting

Cells were collected in RIPA lysis buffer (YEASEN, Shanghai, China) with 1/100 PMSF (YEASEN), centrifuged at 15,000 g at 4℃ for 10 min, and the supernatant was collected. Protein concentrations were determined using a BCA protein assay kit (YEASEN) and samples were diluted to 1.25 mg/mL with RIPA, mixed with 5× sample buffer (YEASEN), and heated to 100℃ for 5 min. Samples of equal protein concentration were separated by 10% sodium dodecyl sulfate (SDS) polyacrylamide gel electrophoresis. Western blotting was performed using standard processes. Primary antibodies used were E-cadherin (Proteintech, China), N-cadherin (CST, Danvers, MA, USA), vimentin (Proteintech, Wuhan, China), Snail (Abcam, Cambridge, MA, USA), β-catenin (Abcam), Cyclin E1 (CCNE1; Abcam), and GAPDH (Proteintech); GAPDH was the internal control.

## Cell transfection

The cells were inoculated into 6-well plates and grown to a density of 70%–80%. siRNA targeting the lncRNA gene was transfected into cells using EndoFectin Max transfection reagent (GeneCopoeia, Guangzhou, China) using 2 µg of siRNA per well. After approximately a 4-h incubation, the transfection medium was substituted with 2 mL of fresh complete medium. The siRNA sequences are shown in Table 2.

**Table 2:** The siRNA sequences used in this study.

siRNAs name	Sequence	
	sense(5’−3’)	antisense(5’−3’)
si_lnc-ALX1-2:5 − 1:	GGUGCAGAAAAGCUUGAAGdTdT	CUUCAAGCUUUUCUGCACCdTdT
si_lnc-ALX1-2:5 − 2:	CUGUCAACCUGGUGCAGAAAAGCUU	AAGCUUUUCUGCACCAGGUUGACAG
si_lnc-ALX1-2:7 − 1:	GCUCCAUGGAAAUCUCAACUAdTdT	UAGUUGAGAUUUCCAUGGAGCdTdT
si_lnc-ALX1-2:7 − 2:	GAGUCAAACCAUAUGCUGUdTdT	ACAGCAUAUGGUUUGACUCdTdT
si_lnc-ALX1-2:10 − 1:	CUUCGGAAAAGCGCAGUAUUCdTdT	GAAUACUGCGCUUUUCCGAAGdTdT
si_lnc-ALX1-2:10 − 2:	GCAGAAAUCACCCGUCUUAdTdT	UAAGACGGGUGAUUUCUGCdTdT
Si-NC	UUCUCCGAACGUGUCACGUdTdT	ACGUGACACGUUCGGAGAAdTdT

## Construction of lnc-ALX1-2:10 knockdown cell line

Approximately 50–100 PC-3 M-1E8 cells were inoculated into a 6-well plate per well; 12 h later, the lentivirus containing sh_lnc-ALX1-2:10 or sh_NC was added into the medium. The sequences were: sh_ lnc-ALX1-2:10, 5′-GCAGAAATCACCCGTCTT‐3′; and sh_NC, 5′‐TTCTCCGAACGTGTCACGT‐3′. After incubation for 72 h, the medium was replaced with fresh medium including puromycin (Sigma, St. Louis, MO, USA) and incubated for another 48 h. The cells were cultured in complete medium with puromycin. The puromycin-resistant cell lines were screened for lnc-ALX1-2:10 expression, and the one with the lowest expression was selected for mRNA sequencing and tumorigenicity experiments.

### Cell proliferation assay

Cells were transfected with siRNA for 4 h. The transfection medium was replaced with fresh medium containing 10% FBS, and cells were further cultured for 12 h. The cells were digested and resuspended into single-cell suspension and then counted (Life technology, Gaithersburg, MD, USA). The cell concentration was diluted to 3 × 10^4^ cells/mL, and the cells were inoculated into 96-well plates, at approximately 3000 cells per well. The cell proliferation assay was performed using the MTS Assay Kit (Abcam), according to the manufacturer’s instructions.

## Transwell migration and invasion assay

Cell migration and invasion abilities were assessed using Transwell chambers (Corning, USA). For the invasion assay, the upper surface of the chamber membrane was pre-coated with 50 µL of Matrigel (Corning) diluted in serum-free medium (1:8 ratio) and allowed to polymerize at 37 °C for 4 h. For the migration assay, chambers were used without Matrigel coating. Cells were transfected with siRNA for 4 h; then, the transfection system was replaced by fresh medium with 10% FBS, and cells were cultured for 12 h. The cells were digested into single-cell suspension and then diluted to 10^6^ cells/mL in DMEM without FBS. Then, 100 µL of cell suspension was added to the upper chamber of a Transwell insert, and 600 µL of complete medium was added to the lower chamber. After culturing, the cells in the upper chamber were wiped, fixed by 4% paraformaldehyde for 15 min, and then stained with 1% crystal violet for 10 min. Cells were observed under a microscope and images captured; the number of cells was counted. The migration rate was defined as the ratio of the number of cells in the experimental group compared with that in the normal control group.

## RNA sequencing

Total RNA was extracted from cells using TRIzol reagent (Invitrogen). RNA integrity and concentration were assessed using an Agilent 2100 Bioanalyzer (Agilent Technologies, USA) and a NanoDrop spectrophotometer (Thermo Fisher Scientific). Only samples with RNA Integrity Number (RIN) ≥ 7.0 were used for subsequent library construction. Library preparation and sequencing were performed as a commercial service by Guangzhou Forevergen Biosciences Co., Ltd. Specifically, ribosomal RNA (rRNA) was removed from approximately 1 µg of total RNA per sample using the Ribo-Zero Gold rRNA Removal Kit (Illumina). The purified RNA was then fragmented and used to construct strand-specific sequencing libraries with the NEBNext Ultra II Directional RNA Library Prep Kit (New England Biolabs, USA) according to the manufacturer‘s protocol. The final cDNA libraries were quantified, and their size distribution was assessed using an Agilent 2100 Bioanalyzer. Paired-end sequencing (2 × 150 bp) was performed on an Illumina NovaSeq 6000 platform to generate at least 20 million raw reads per sample.

For bioinformatic analysis, raw sequencing reads were first processed to ensure data quality. Adapter sequences and low-quality bases (Phred score < 20) were trimmed using Trimmomatic (v0.39). The cleaned reads were then aligned to the human reference genome (GRCh38/hg38) using HISAT2 (v2.2.1). Transcript assembly and quantification were performed using StringTie (v2.1.5) with the GENCODE (v35) annotation as a reference. The transcript abundance for each gene was estimated as Fragments Per Kilobase of transcript per Million mapped reads (FPKM). Differential expression analysis between the two experimental groups (e.g., sh_NC (negative control) vs. sh_lnc-ALX1-2:10) was conducted using the edgeR package (v3.28.1) based on read counts in R, with genes showing an absolute fold change ≥ 2 and an adjusted p-value (FDR) < 0.05 considered significantly differentially expressed. Gene Ontology (GO) and Kyoto Encyclopedia of Genes and Genomes (KEGG) pathway enrichment analyses for the differentially expressed genes were performed using the clusterProfiler package (v4.0.5) in R. The primary bioinformatic processing (quality control, alignment, and quantification) was provided by the service provider, while the subsequent differential expression analysis and functional enrichment interpretation were conducted by the authors.

### Tumorigenicity experiment in nude mice

A total of 13 BALB/c nude mice (5–6 weeks old, specific pathogen free grade) were randomly divided into two groups using a random number table: sh_NC group (*N* = 6 housed in one cage with the situation of specific pathogen free grade), sh_lnc-ALX1-2:10 group (*N* = 7 cultured in one cage with the situation of specific pathogen free grade). PC-3 M-1E8 sh_NC and PC-3 M-1E8 sh_lnc-ALX1-2:10 cells were injected subcutaneously into the dorsal thighs of the mice. The tumor volume and body weight were observed every week for 5 weeks, in which the tumor volume was calculated as follows: length × width^2^ × 0.5. When the tumor volume was greater than 2000 mm^3^, weight loss exceeded 20%, or the mice were unable to obtain food and water independently, euthanasia was implemented. There were no animals meeting the criteria for euthanasia before the collection of tumor tissues. After 5 weeks, all mice were anesthetized with 3% isoflurane inhalation and euthanized with CO_2_. The initial CO_2_ flow rate was approximately 70% V/min. All animal euthanasia procedures conform to American Veterinary Medical Association (AVMA) Guidelines for the Euthanasia of Animals: 2020 Edition. Animal experiments were conducted by Guangzhou Forevergen Biosciences Co., Ltd. as a commercial service. The tumor tissue was collected for further analysis.

### Statistical analysis

All data analysis, except RNA sequencing, used GraphPad Prism 7.0 (GraphPad Software, Inc.,La Jolla, CA, USA) and was presented as mean ± SEM. Statistical analysis was conducted by either Student’s t-test (two-group comparison) or one-way analysis of variance (more than two groups), asterisks represent the following: *P < 0.05, **P < 0.01, ***P < 0.001 and ****P < 0.0001.

## Results

### lnc-ALX1-2 gene cluster was highly expressed in the PC-3 M-1E8 PCa cell line

**Fig. 1:**
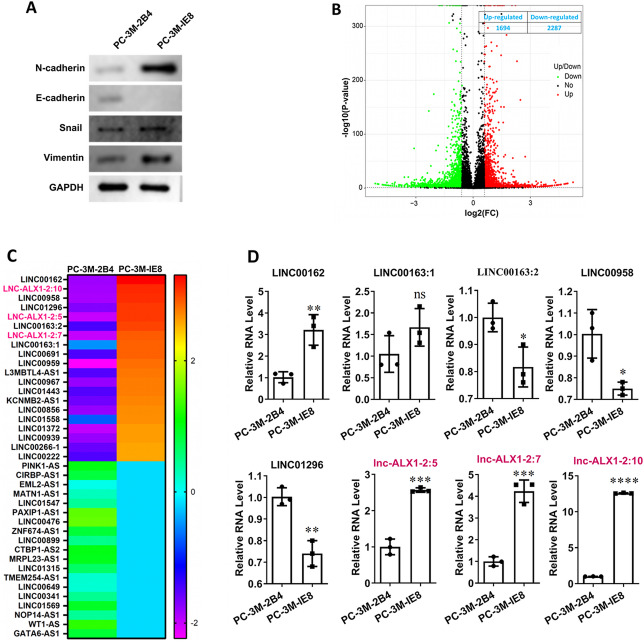
The expression of the lnc-ALX1-2 transcript cluster was upregulated in the highly metastatic prostate cancer (PCa) cell line. (**A**) EMT-associated proteins (N-cadherin, E-cadherin, Snail, and vimentin) were assayed by western blot. The original blots are presented in Supplementary Fig. 1. (**B**) Scatter plot shows the differential expression of molecules from complete transcriptome sequencing (focusing only on lncRNAs) in both PC-3 M-1E8 and PC-3 M-2B4 cells. (**C**) The heatmap displays the 40 lncRNAs with the largest difference in expression between PC-3 M-1E8 and PC-3 M-2B4 cell lines: 20 were upregulated and 20 were downregulated. (**D**) The expression levels of eight lncRNAs selected from the above heatmap were confirmed by RT-qPCR.

To explore the role of lncRNAs in the proliferation, invasion, and migration of PCa cells, we selected the highly metastatic PCa cell line (PC-3 M-1E8) and the poorly metastatic PCa cell line (PC-3 M-2B4) and then compared the differences in lncRNA and protein expression between these two cell lines. The expression levels of N-cadherin, Snail, and vimentin were significantly higher, while the expression level of E-cadherin was significantly lower in PC-3 M-1E8 cells compared to those in PC-3 M-2B4 cells, which confirmed the expected EMT phenotype associated with the higher metastatic potential of PC-3 M-1E8 cells compared to PC-3 M-2B4 cells (Fig. 1A). We analyzed the above two cell lines using the whole transcriptome and focused on lncRNA expression. There were 1694 upregulated lncRNAs and 2287 downregulated lncRNAs in PC-3 M-1E8 cells compared with those in PC-3 M-2B4 cells (Fig. 1B). Among these lncRNAs, 40 (the top 20 upregulated and top 20 downregulated) were selected for display (Fig. 1C). The top eight upregulated lncRNAs formed a prominent group, and their expression was confirmed by RT-qPCR. The expression levels of three members of the lnc-ALX1-2 cluster were upregulated in PC-3 M-1E8 cells compared with those in PC-3 M-2B4 cells (Fig. 1D). To summarize, the lnc-ALX1-2 gene cluster was highly expressed in the highly metastatic PCa cell line.

### The knockdown of lnc-ALX1-2 gene cluster suppressed proliferation, migration and invasion in a highly metastatic PCa cell line

**Fig. 2:**
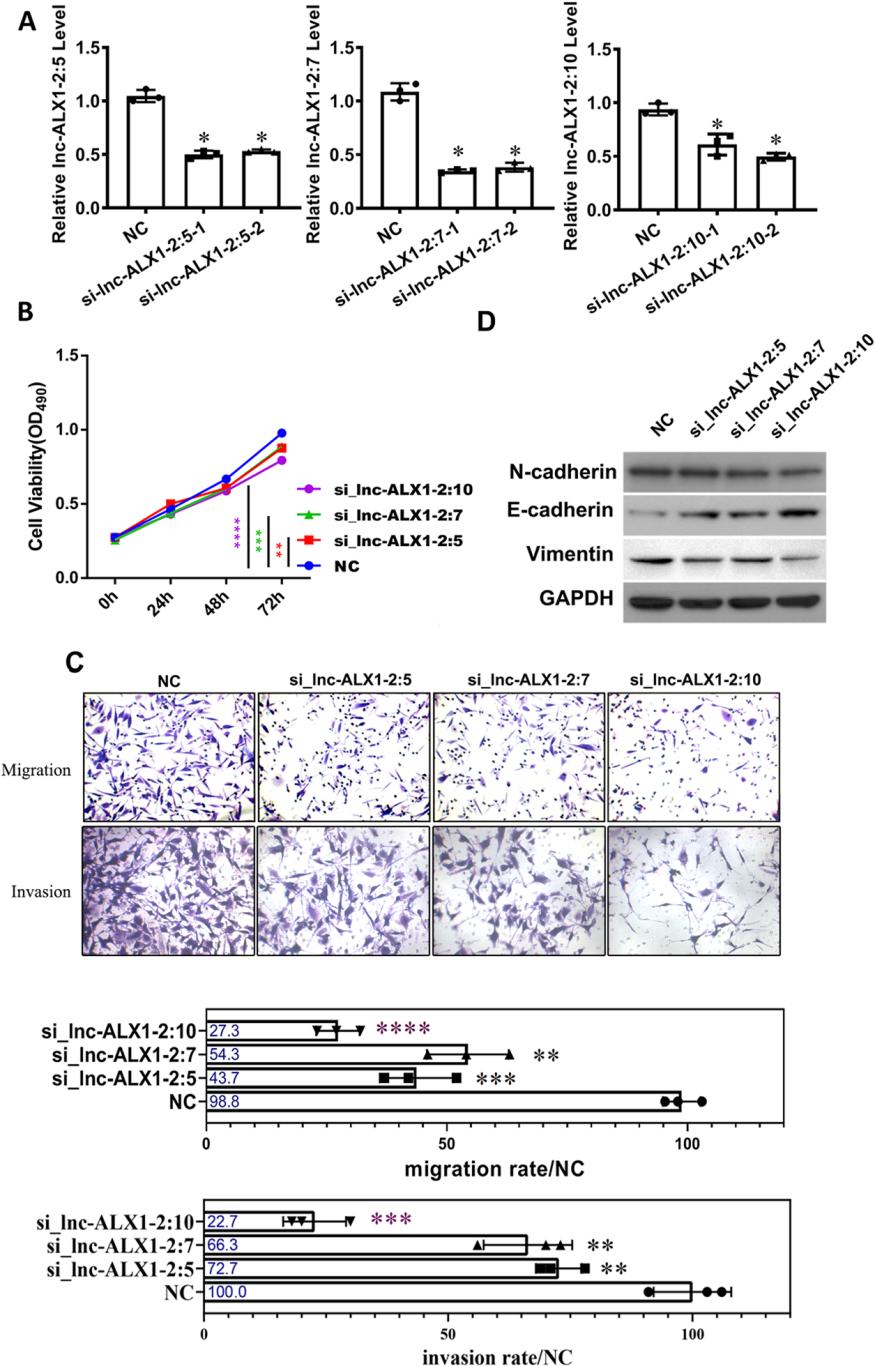
The knockdown of the lnc-ALX1-2 cluster reduced proliferation, migration, invasion, and EMT of PC-3 M-1E8 cells. (**A**) The knockdown of the three members of the lnc-ALX1-2 cluster was performed by siRNA, and the knockdown efficiency was detected by RT-qPCR. (**B**) The proliferation ability of PC-3 M-1E8 cells after siRNA transfection was tested by MTS assay. (**C**) Transwell assay was performed on PC-3 M-1E8 cells after siRNA transfection, and the results are shown by photos and statistics. (**D**) The protein level expression of N-cadherin, E-cadherin, and vimentin in PC-3 M-1E8 cells after siRNA transfection was detected. The original blots are presented in Supplementary Fig. 2.

The above results indicated that there was a possible connection between the upregulated expression of the lnc-ALX1-2 gene cluster and the high metastatic potential of the PC-3 M-1E8 cell line. We designed siRNA sequences to target the three members of the lnc-ALX1-2 cluster and tested their knockdown efficiency in PC-3 M-1E8 cells (Fig. 2A). The siRNA sequences with the highest knockdown efficiency were used for subsequent experiments. The cell proliferation efficiency in si-lnc-ALX1-2 cluster knockdown groups was significantly lower than that in the si-NC (siRNA negative control) group. Specifically, the knockdown effect was most prominent in the si-lnc-ALX1-2:10 group (Fig. 2B). Transwell analysis indicated that the migration and invasion abilities of si-lnc-ALX1-2-treated cells were significantly inhibited compared with those of the si-NC-treated group. In particular, the number of cells was most reduced in the si-lnc-ALX1-2:10-treated group (Fig. 2C). The expression levels of N-cadherin and vimentin were downregulated, while the expression level of E-cadherin was increased in the si-lnc-ALX1-2:10 group in comparison with those in the si-NC group, suggesting that the epithelial mesenchymal transition (EMT) was suppressed in this group (Fig. 2D). In short, the proliferation and migration of PCa cells and the expression of EMT markers could be largely decreased by the knockdown of the lnc-ALX1-2 gene cluster, of which the knockdown of lnc-ALX1-2:10 was the most effective.

### The alteration of mRNA expression induced by lnc-ALX1-2:10 knockdown in a highly metastatic PCa cell line

**Fig. 3:**
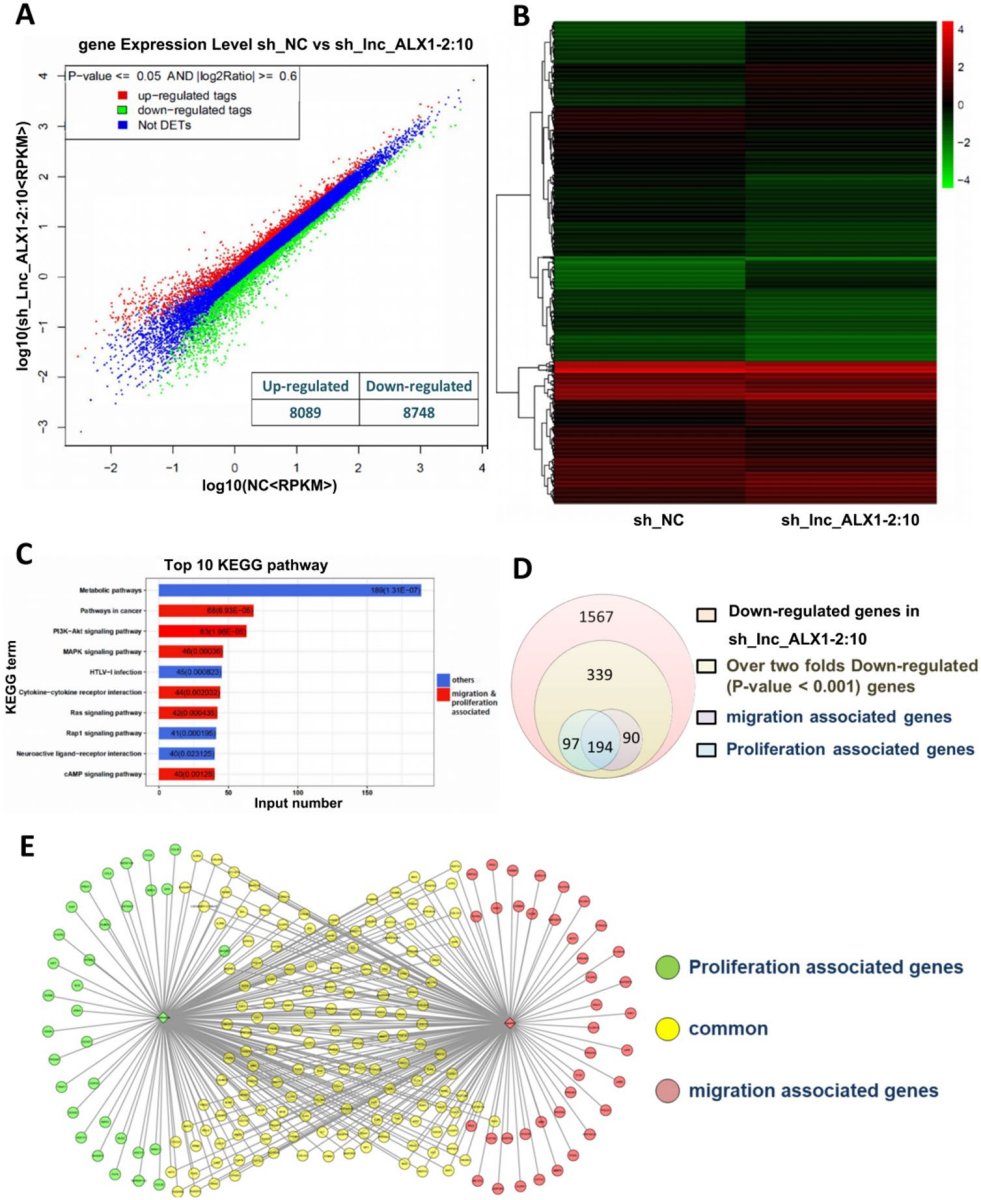
Analysis of mRNA expression profile by complete transcriptome sequencing. (**A**) Scatter plot of mRNA from transcriptome sequencing in PC-3 M-1E8 cells transfected with either sh_NC or sh_lnc-ALX1-2:10. (**B**) The differential mRNA expression profile between PC-3 M-1E8 cells transfected with either sh_NC or sh_lnc-ALX1-2:10 is shown by a heatmap. (**C**) The top 10 pathways associated with the differential expression of mRNA in KEGG pathway enrichment analysis results. The red color indicates the pathways related to proliferation and migration. KEGG analysis was conducted with the permission of Kanehisa laboratory^20–22^. (**D**) Venn diagram shows the downregulated genes. (**E**) The network of migration- and proliferation-associated genes is illustrated according to the protein interaction relationships of mRNAs.

We constructed a PC-3 M-1E8 cell line with stable knockdown of lnc-ALX1-2:10 and analyzed the differences in mRNA expression between the PC-3 M-1E8 cell line with stable knockdown of lnc-ALX1-2:10 and PC-3 M-1E8 cells containing an empty vector. The expression of 8089 genes was upregulated, while that of 8748 genes was downregulated in cells with stable knockdown of lnc-ALX1-2:10 (Fig. 3A). Cluster analysis showed significant differential mRNA expression between the two groups (Fig. 3B). All differentially expressed mRNAs were subjected to Kyoto Encyclopedia of Genes and Genomes (KEGG) pathway enrichment analysis to identify related pathways, and the top 10 pathways are shown in Fig. 3C. Six of the first 10 signaling pathways were related to cell proliferation and migration, including signaling pathways in cancer, PI3K-Akt, MAPK, cytokine-cytokine receptor interaction, Ras, and cAMP. We then focused on the downregulated genes induced by lnc-ALX1-2:10 knockdown. The expression levels of 720 genes in the lnc-ALX1-2:10 knockdown group were twofold lower than those in the control group. Among these genes, 284 were related to migration, 291 were related to proliferation, and 194 were involved in both migration and proliferation (Fig. 3D). Finally, the protein interactions of the above genes were described in detail and visualized (Fig. 3E).

**Fig. 4:**
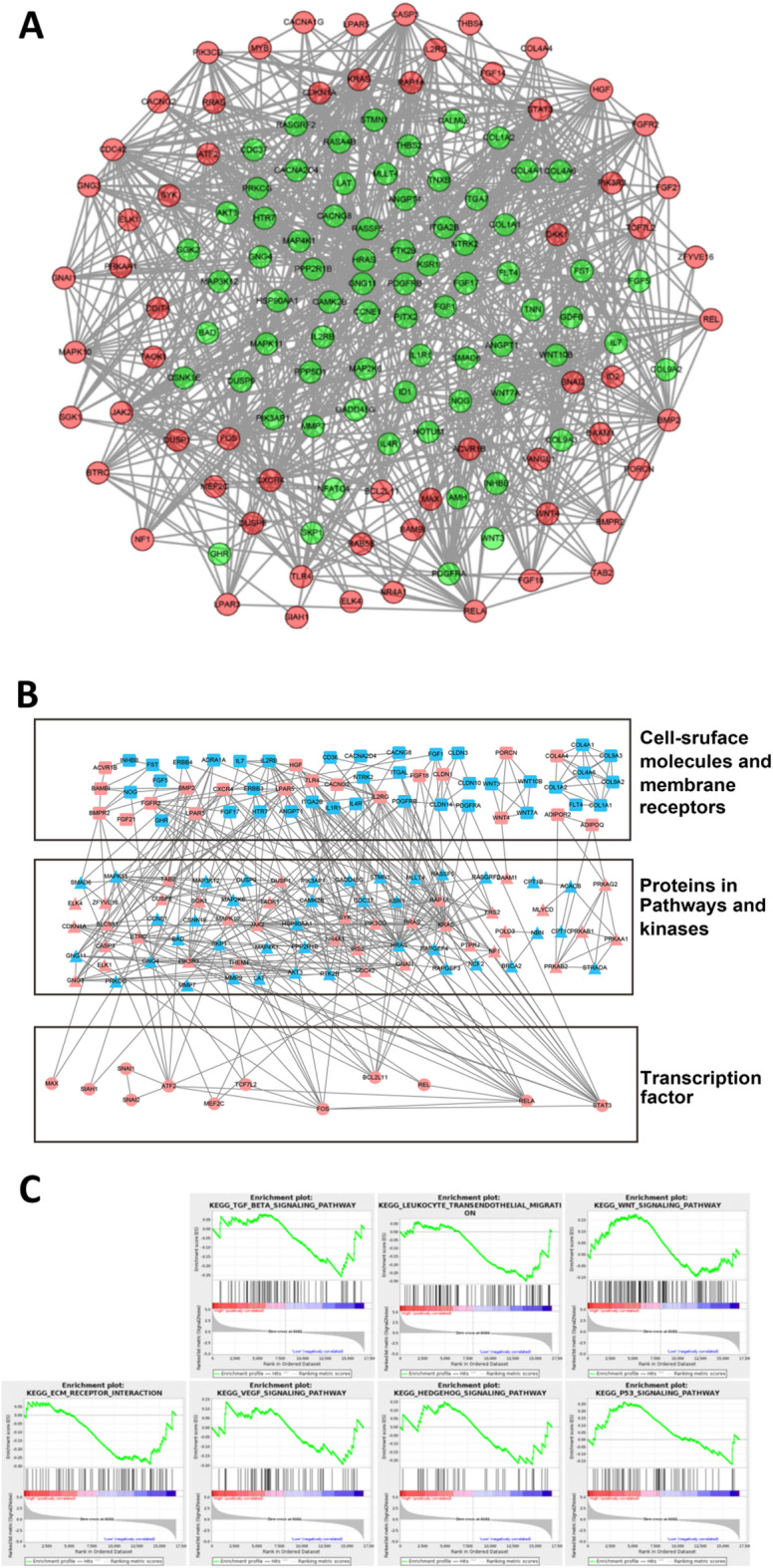
Network of proliferation- and migration-associated genes induced by lnc-ALX1-2:10 knockdown. (**A**) According to the protein-protein interaction information from the KEGG database, the protein interaction network related to proliferation and migration was delineated in PC-3 M-1E8 cells with lnc-ALX1-2:10 knockdown; red represents the upregulated genes, and green represents the downregulated genes. (**B**) The molecular network of differential mRNA expression related to proliferation and migration between PC-3 M-1E8 cells transfected with siRNA-NC or with si-lnc-ALX1-2:10 was constructed at multiple levels, based on the protein KEGG database. Red dots are the upregulated genes, and blue dots are the downregulated genes; top panel represents cell surface molecules or membrane receptors, middle panel represents downstream kinase-related pathway molecules, and lower panel represents transcription factors. (**C**) The regulated models of lnc-ALX1-2:10 knockdown or normal expression in signal pathways involved in proliferation and migration were shown by gene set enrichment analysis.

We also analyzed and visualized the protein-protein interaction of genes that were altered by lnc-ALX1-2:10 knockdown and that were related to migration and proliferation. Genes with downregulated expression accounted for the majority of the genes that were altered by lnc-ALX1- 2:10 knockdown and related to migration and proliferation, and most of these were in the center of the molecular regulatory network (Fig. 4A). Based on the pathway information from the KEGG database related to proteins, 194 genes related to migration and proliferation in the lnc-ALX1-2:10-knockdown PC-3 M-1E8 cells were used to construct a protein interaction network according to the location and function of their corresponding proteins in cells (Fig. 4B). In the three levels of this network (cell-surface molecules and membrane receptors; proteins in pathways and kinases; transcription factors), the molecular networks were concentrated on a few genes. Furthermore, notably, all the molecular networks were concentrated on a few transcription factors, particularly signal transducer and activator of transcription 3 (STAT3), RELA, BCL2-like 11 (BCL2L11), FOS, and activating transcription factor 2 (ATF2). Gene set enrichment analysis (GSEA) was performed to explore the effects of lnc-ALX1-2:10 knockdown on the regulation of signaling pathways involving proliferation and migration; the results showed that lnc-ALX1-2:10 knockdown negatively regulated the extracellular matrix (ECM), transforming growth factor beta (TGF-β), leukocyte transendothelial migration, and Hedgehog pathways but positively regulated the WNT and p53 pathways (Fig. 4C). Thus, the knockdown of lnc-ALX1-2:10 expression could significantly regulate multiple genes related to proliferation and migration by modulating the expression of only a few transcription factors.

### Validation of proliferation and migration associated with major genes affected by lnc-ALX1-2:10 knockdown in a highly metastatic PCa cell line

**Fig. 5:**
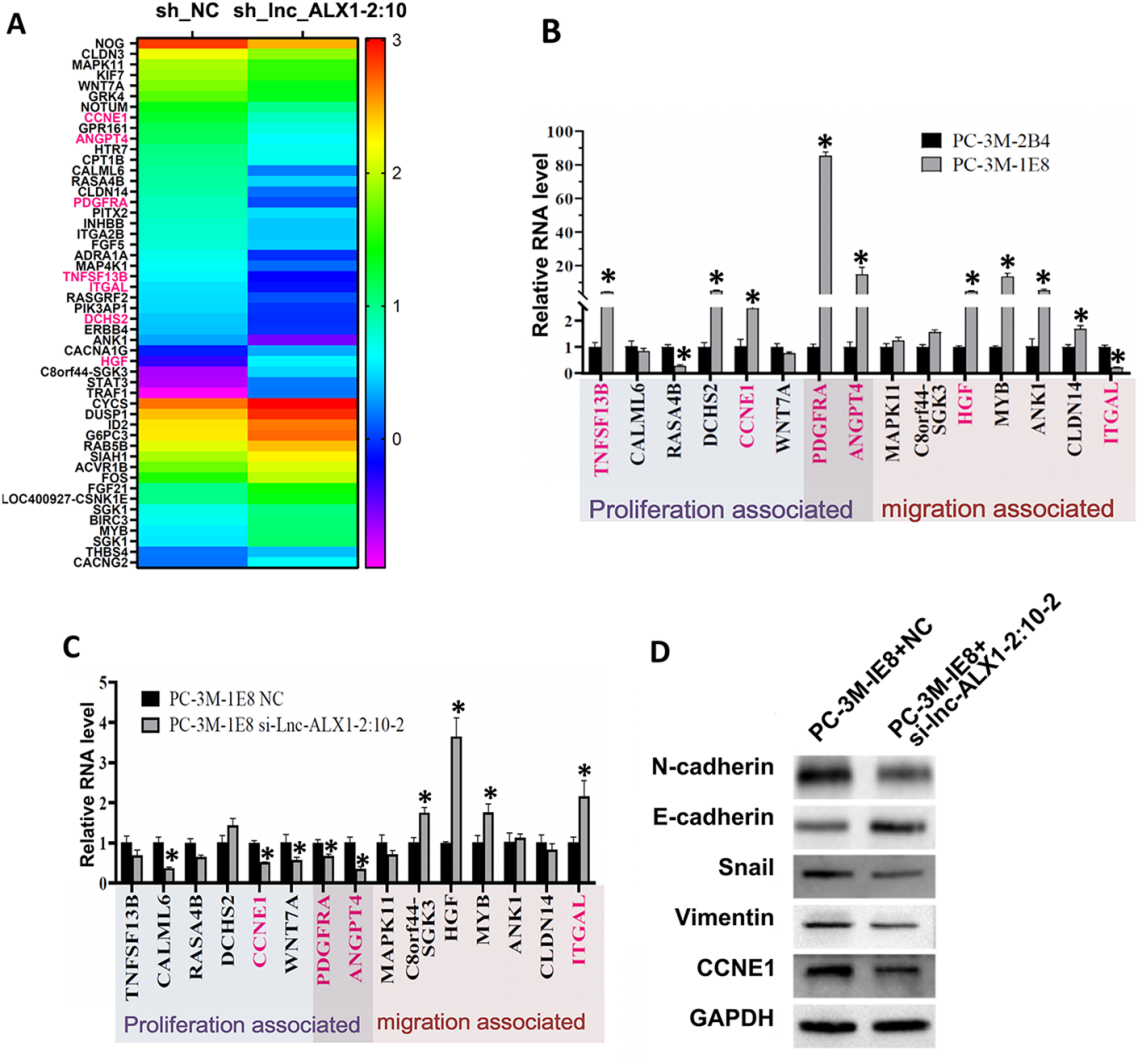
Verification of key genes associated with proliferation and migration regulated by lnc-ALX1-2:10 in PC-3 M-1E8 cells. (**A**) A heatmap was generated to display 50 genes (top 29 highly expressed in the normal control group and top 21 highly expressed in the lnc-ALX1-2:10 knockdown group) selected from a pool of 194 genes associated with proliferation and migration. (**B**) The expression of key molecules in the heatmap was verified by RT-qPCR in PC-3 M-2B4 and PC-3 M-1E8 cells. (**C**) The expression of key molecules in the heatmap was verified by RT-qPCR in PC-3 M-1E8 cells transfected with siRNA-NC or si-lnc-ALX1-2:10. (**D**) The expression levels of EMT proteins and cell proliferation-related molecules at protein level were analyzed in PC-3 M-1E8 cells transfected with siRNA-NC or si-lnc-ALX1-2:10. The original blots are presented in Supplementary Fig. 3.

The above results indicated that lnc-ALX1-2:10 knockdown could significantly affect the expression of genes related to proliferation and migration in PC-3 M-1E8 cells. We drew a molecular expression heatmap by selecting 50 genes related to cell proliferation and migration. Among these 50 genes, 29 were from the top-expressed genes in the normal control group and 21 were from the top-expressed genes in the lnc-ALX1 −2:10 knockdown group (Fig. 5A). Sixteen representative genes were selected and verified by RT-qPCR, most of which were consistent with the results shown in the heatmap (Fig. 5B and C). Finally, Western blot analysis revealed that knockdown of lnc-ALX1-2:10 in PC-3 M-1E8 cells significantly reduced the expression of EMT markers (N-cadherin, Snail, and vimentin) and cell proliferation-related genes (CCNE1). Meanwhile, the epithelial marker E-cadherin was significantly upregulated (Fig. 5D). Thus, lnc-ALX1-2:10 knockdown in PCa cells may regulate multiple genes related to cell proliferation and migration.

### Validation of the proliferation and migration associated with major genes affected by lnc-ALX1-2:10 knockdown in vivo

**Fig. 6:**
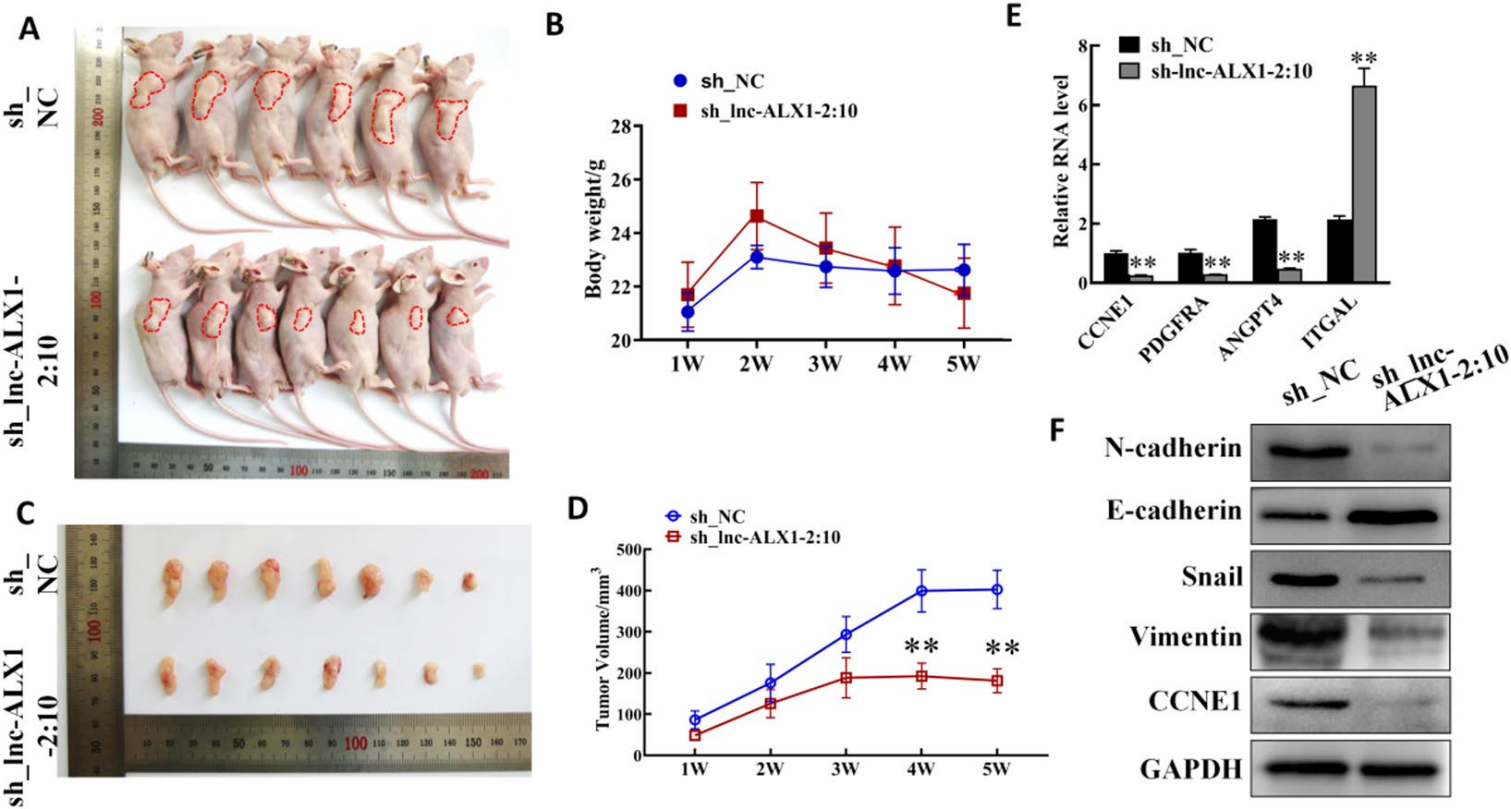
Validation of the proliferation and migration associated with major genes affected by lnc-ALX1-2:10 knockdown in vivo. (**A**) The photographs of nude mice were captured in the fifth weeks after cell injection into the mice (NC group, *N* = 6; sh-lnc-ALX1-2:10 group, *N* = 7). (**B**) The changes in the body weight of mice over five weeks after cell injection into the mice are presented in a line chart. (**C**) Photographs of tumor tissues were captured in the fifth weeks after cell injection into the mice. (**D**) The changes in tumor volume over five weeks after cell injection into the mice are shown in a line chart. (E) RT-qPCR was used to evaluate the expression levels of CCNE1, PDGFRA, ANGPT4, and ITGAL in tumor tissues. (**F**) Western blot analysis was used to detect the expression levels of N-cadherin, E-cadherin, Snail, Vimentin, and CCNE1 in tumor tissues. The original blots are presented in Supplementary Fig. 4.

We further investigated its role in vivo. As shown in Fig. 6A and D, lnc-ALX1-2:10 knockdown significantly decreased tumor volume but had no effect on the body weights of mice. In addition, the expression of proliferation- and migration-related genes was also detected in tumor tissues using RT-qPCR and Western blot analysis. Results showed that the expression levels of CCNE1, PDGFRA, ANGPT4, N-cadherin, Snail, and Vimentin were all suppressed, while the expression levels of E-cadherin and ITGAL were promoted after lnc-ALX1-2:10 knockdown compared to those in the sh_NC group (Fig. 6E and F). These results were consistent with the in vitro findings.

## Discussion

PCa remains a major clinical challenge due to its high metastatic potential and limited therapeutic options for advanced disease^23^. LncRNAs have emerged as critical regulators in PCa progression, with increasing evidence supporting their roles as biomarkers and therapeutic targets^10–12^. To identify novel metastasis-associated lncRNAs, we performed transcriptome sequencing of isogenic PCa cell lines with divergent metastatic potential (PC-3 M-1E8 vs. PC-3 M-2B4). This screening identified the lnc-ALX1-2 gene cluster as significantly upregulated in the highly metastatic cells. Notably, this lncRNA (also annotated as LINC02820) has been implicated in other malignancies, exhibiting pro-invasive functions in bladder and esophageal squamous cell carcinomas^13–15^. Its genomic neighbor, the transcription factor ALX1, also drives invasion and EMT in cancers such as lung and ovarian cancer^16,17^. Our study extends the functional relevance of this RNA to PCa, being the first to demonstrate that knockdown of its specific transcript, lnc-ALX1-2:10, is associated with the suppression of proliferation, migration, invasion, and in vivo tumor growth, concomitant with molecular shifts indicative of attenuated EMT and altered oncogenic signaling. Furthermore, our screening set included other lncRNAs known to play roles in cancer progression, such as LINC00958, which enhances migration and invasion in bladder cancer^13^; LINC01296, linked to poor prognosis and promoting PCa proliferation and metastasis^24^; and LINC00162, associated with sorafenib resistance in thyroid cancer^25^. This co-enrichment further confirms the reliability of our model in identifying functionally important transcripts and suggests the existence of common oncogenic pathways across different cancer types.

RNA-seq analysis upon lnc-ALX1-2:10 knockdown revealed broad transcriptional reprogramming, associated with the downregulation of 194 genes linked to proliferation and migration. Pathway enrichment highlighted perturbations in core oncogenic cascades such as PI3K-Akt and MAPK signaling, which are well-established drivers of PCa progression^26,27^. Notably, the downregulation of PDGFRA and CCNE1—key genes at the center of our interaction network—resonates with prior studies showing that the PDGF/PDGFR axis promotes EMT and bone metastasis in PCa^28^, and that CCNE1 enhances cancer cell survival^29^. These concordances suggest that lnc-ALX1-2:10 may intersect with established PCa pathways. Network analysis further indicated that these widespread changes might converge on central transcription factors like STAT3, whose role in PCa is complex and context-dependent, involved in both promotion and suppression of malignancy^30–32^. It is crucial to emphasize that our data establish an association between lnc-ALX1-2:10 knockdown and these molecular changes; definitive mechanistic causality awaits future interrogation.

Despite these intriguing observations, this study has several notable limitations that must be addressed in future work. First, the findings are primarily based on a single, well-characterized yet limited cell line model (PC-3 M-1E8/2B4) representing metastatic heterogeneity. The broader applicability of lnc-ALX1-2:10’s function across diverse PCa molecular subtypes necessitates validation using a more extensive panel of cell lines. Second, and most crucially for clinical relevance, data on lnc-ALX1-2:10 expression in human tissue samples are lacking. It remains imperative to assess whether this lncRNA is upregulated in prostate tumors compared to benign tissue and whether its expression correlates with aggressive pathological features or patient prognosis. Third, the exact molecular mechanism—whether transcriptional, post-transcriptional, or epigenetic—remains unresolved. Finally, while our in vivo data demonstrate a role in primary tumor growth, direct evidence for its function in metastasis is lacking. Future studies using specialized metastasis models, e.g., tail vein injection, are needed.

## Conclusions

In conclusion, our findings reveal that lnc-ALX1-2:10 is a novel lncRNA associated with the proliferation, migration, and invasion of PCa cells. This work provides the first functional evidence for the lnc-ALX1-2 locus in PCa, establishing lnc-ALX1-2:10 as a promising target for further mechanistic and translational research to address metastatic progression.

## Supplementary Information

Below is the link to the electronic supplementary material.

Supplementary Material 1

## Data Availability

The datasets generated by Forevergen Biosciences are available in NCBI with the ID: PRJNA753212(https://www.ncbi.nlm.nih.gov/bioproject/PRJNA753212).
