# Supplementary material for: lnc-ALX1-2:10 is a novel regulator that enhances proliferation, migration and invasion in prostate cancer cells
Source: Sci Rep. 2026 Mar 3;16:11870. doi: 10.1038/s41598-026-42299-0 (PMC13065844; doi:10.1038/s41598-026-42299-0)
Supplement: Supplementary file 1 — Supplementary Material 1 [file 41598_2026_42299_MOESM1_ESM.pdf]

lnc-ALX1-2:10 is a novel regulator that enhances proliferation, migration and invasion in prostate cancer cells

Running title: lnc-ALX1-2:10 promotes prostate cancer progression

Xinjun Wang[1,2,#], Qian Zong[2,1#], Yue Bu[1], Bin Zhou[2,1], Xuqiang Wang[1], Zhangqun Li[1]， Guangcheng Luo[1,2, *]

1Department of Urology, Zhongshan Hospital Xiamen University, School of Medicine, Xiamen University, Xiamen, 361004, China
2The School of Clinical Medicine, Fujian Medical University, Fuzhou, 350122, China

#Co-first author:
Xin-Jun Wang and Qian Zong contributed equally to this work.

*Corresponding author:
Guangcheng Luo
Zhongshan Hospital Xiamen University. No.209 Hubin South Road, Xiamen, 361004, China
Tel: +86-0592-2993110
Fax: +86-0592-2212328
Email: lgch@xmu.edu.cn

Figure 1

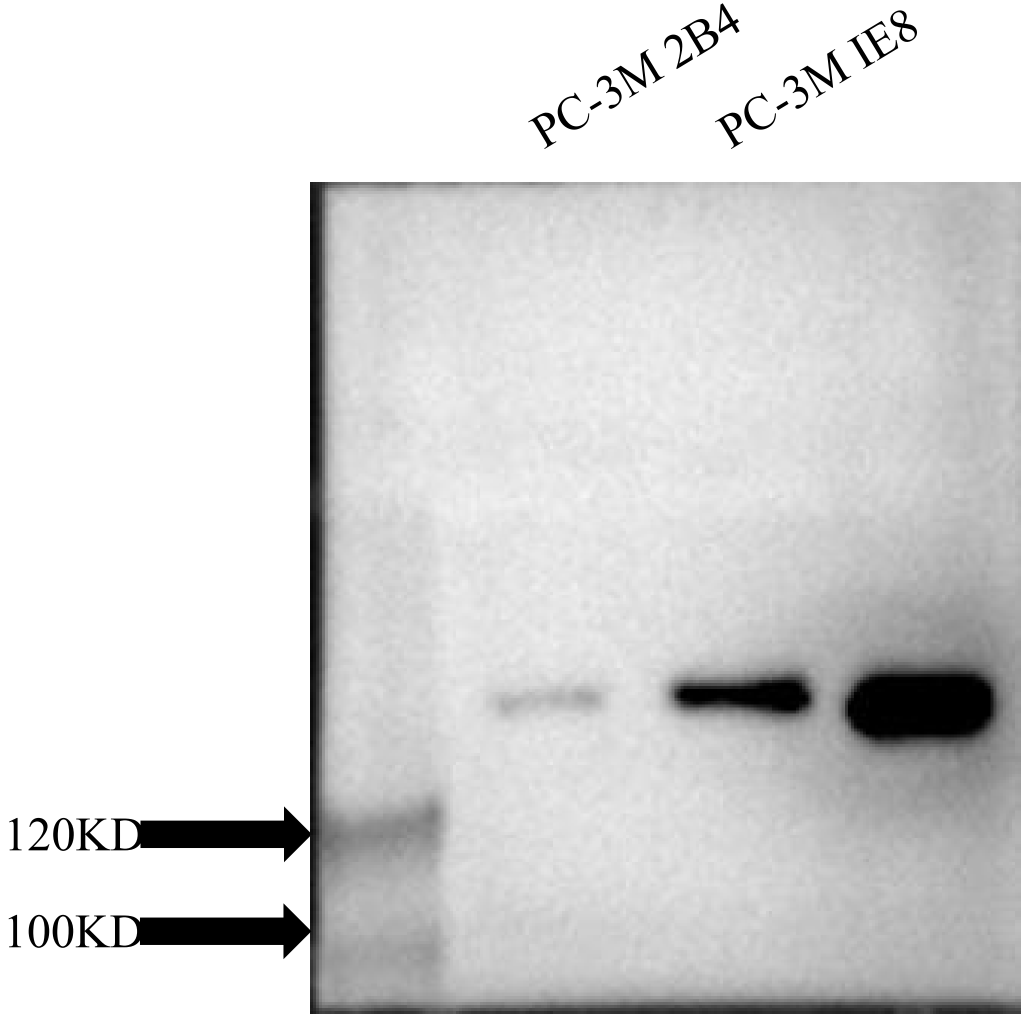

Anti-N Cadherin, santa, sc53488, 1:500, 130KD
HRP-conjugated Affinipure Goat Anti-Mouse IgG(H+L), Proteintech, #SA00001-1; RRID, AB_2722565; 1:1000

Blue Plus II Protein Marker (14-120 kDa), Transgen, #DM111-02

Figure 1

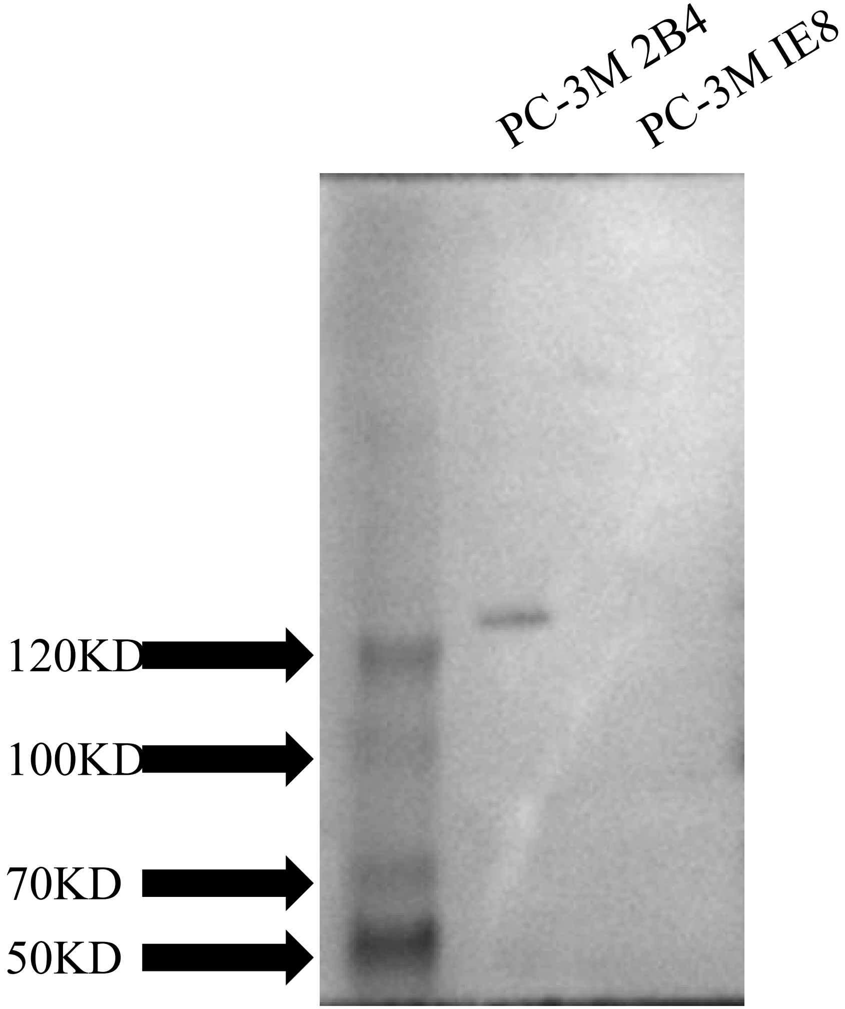

E-cadherin Polyclonal antibody, Proteintech, #20874-1-AP; RRID, AB_10697811; 1:4000, 120-125KD
HRP-conjugated Affinipure Goat Anti-Rabbit IgG(H+L), Proteintech, # SA00001-2; RRID, AB_27225641; 1:5000

Blue Plus II Protein Marker (14-120 kDa), Transgen, #DM111-02

Figure 1

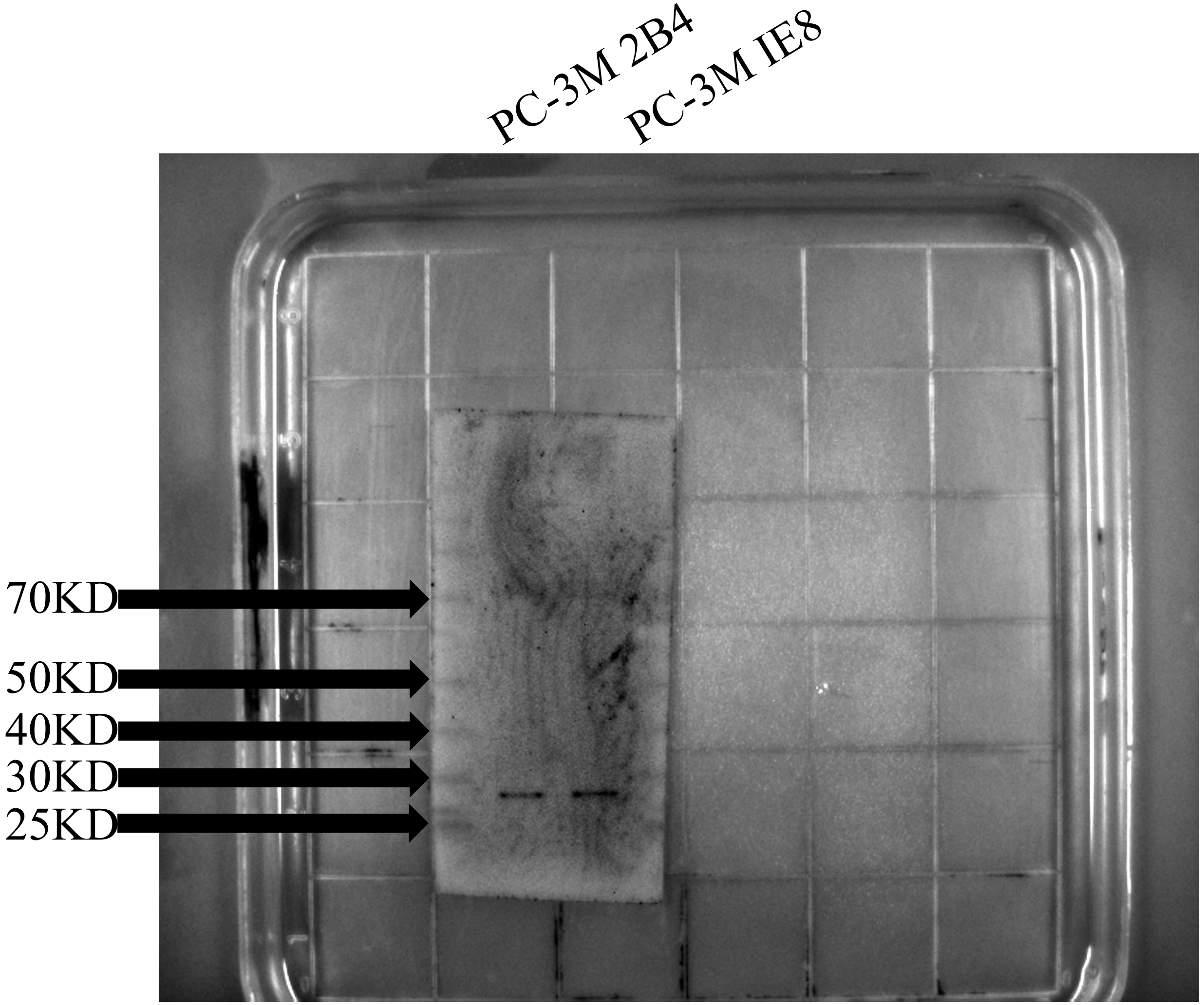

SNAI 1 (G-7), Santa, #sc-271977, 1:1000, 29KD
HRP-conjugated Affinipure Goat Anti-Mouse IgG(H+L), Proteintech, #SA00001-1; RRID, AB_2722565; 1:1000

Blue Plus II Protein Marker (14-120 kDa), Transgen, #DM111-02

Figure 1

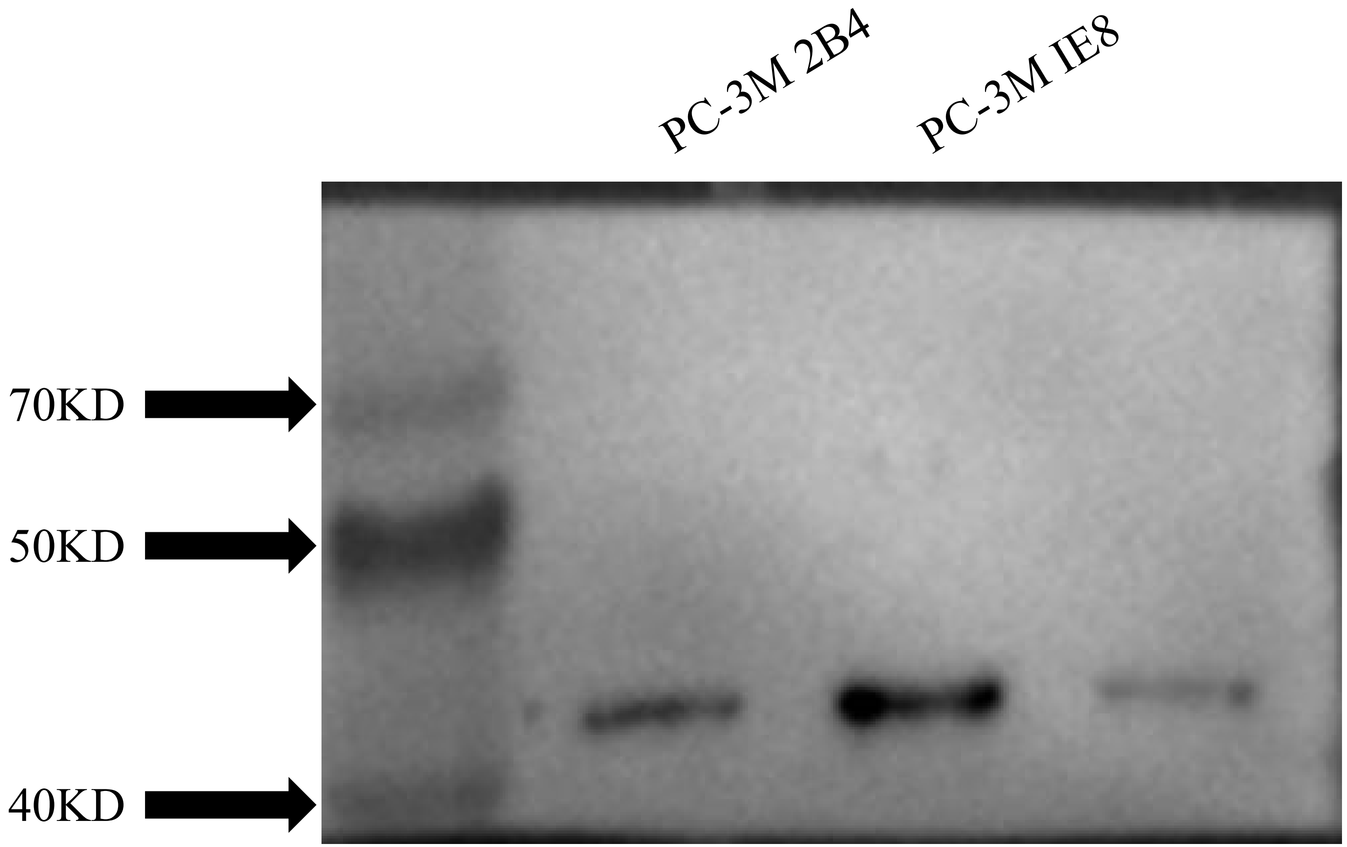

Vimentin (V9), Santa, #sc-6260, 1:500, 43-57KD
HRP-conjugated Affinipure Goat Anti-Mouse IgG(H+L), Proteintech, #SA00001-1; RRID, AB_2722565; 1:1000

Blue Plus II Protein Marker (14-120 kDa), Transgen, #DM111-02

Figure 1

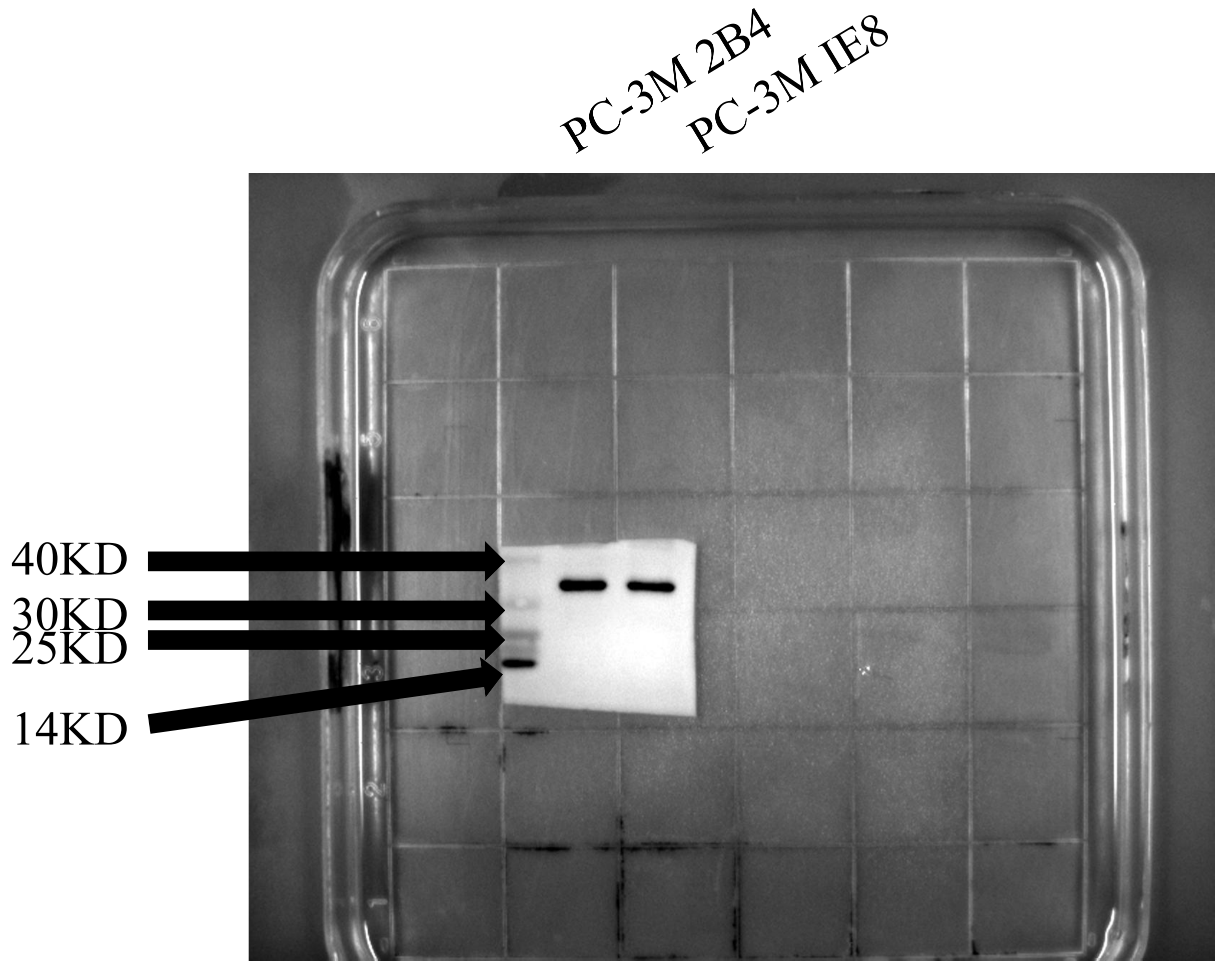

GAPDH Monoclonal antibody, Proteintech, #60004-1-Ig, RRID, AB_2107436; 1:10000, 36KD
HRP-conjugated Affinipure Goat Anti-Mouse IgG(H+L), Proteintech, #SA00001-1; RRID, AB_2722565; 1:5000

Blue Plus II Protein Marker (14-120 kDa), Transgen, #DM111-02

Figure 2

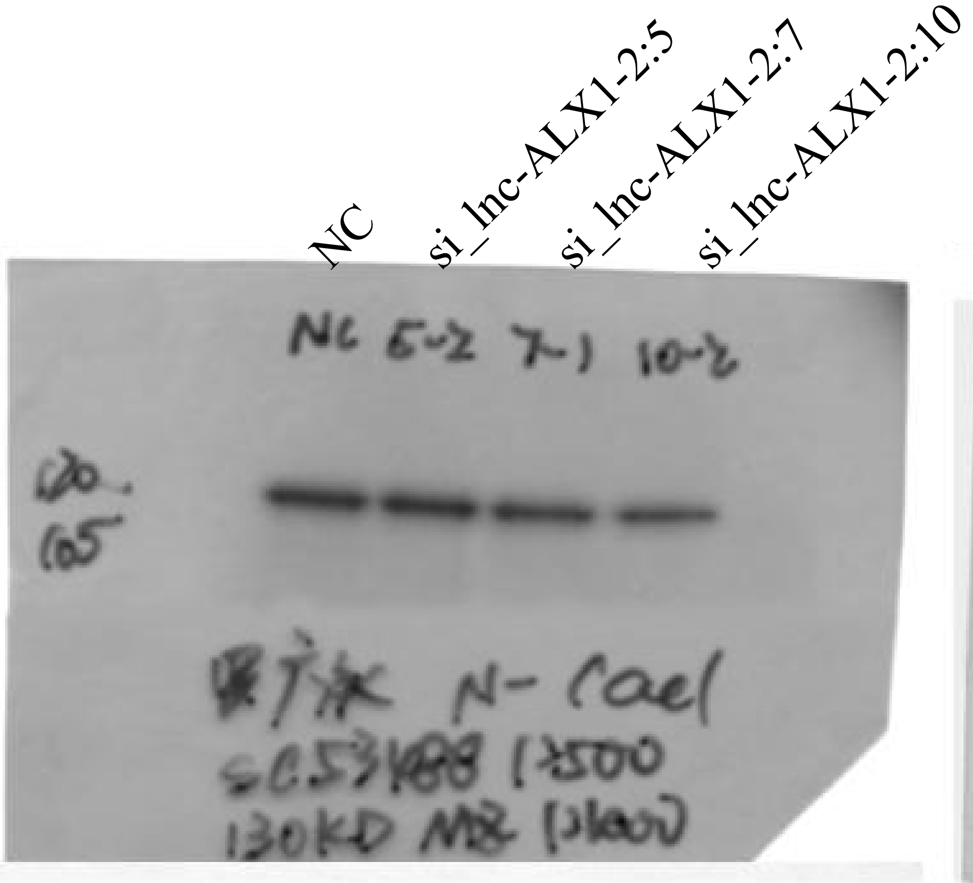

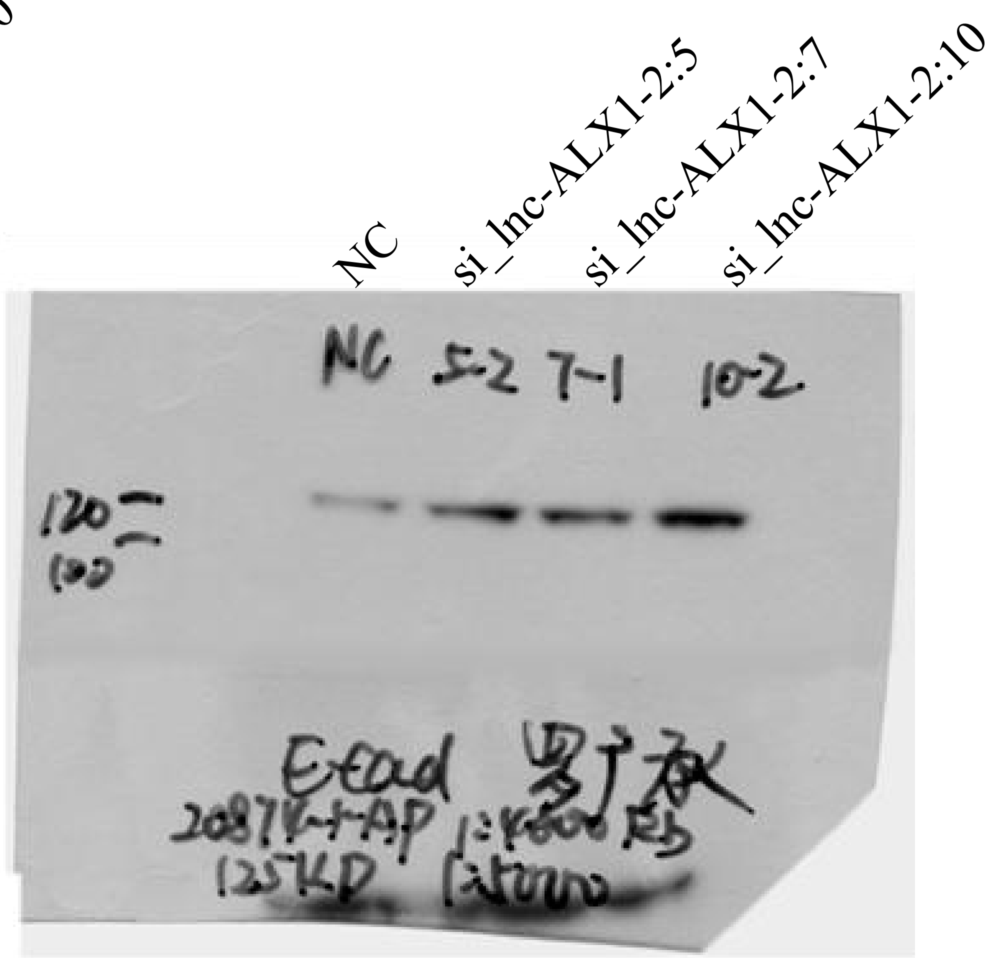

Left
Anti-N Cadherin, santa, sc53488, 1:500, 130KD
HRP-conjugated Affinipure Goat Anti-Mouse IgG(H+L), Proteintech, #SA00001-1; RRID, AB_2722565; 1:1000
Right
E-cadherin Polyclonal antibody, Proteintech, #20874-1-AP; RRID, AB_10697811; 1:4000, 120-125KD
HRP-conjugated Affinipure Goat Anti-Rabbit IgG(H+L), Proteintech, # SA00001-2; RRID, AB_27225641; 1:5000

Blue Plus II Protein Marker (14-120 kDa), Transgen, #DM111-02

Figure 2

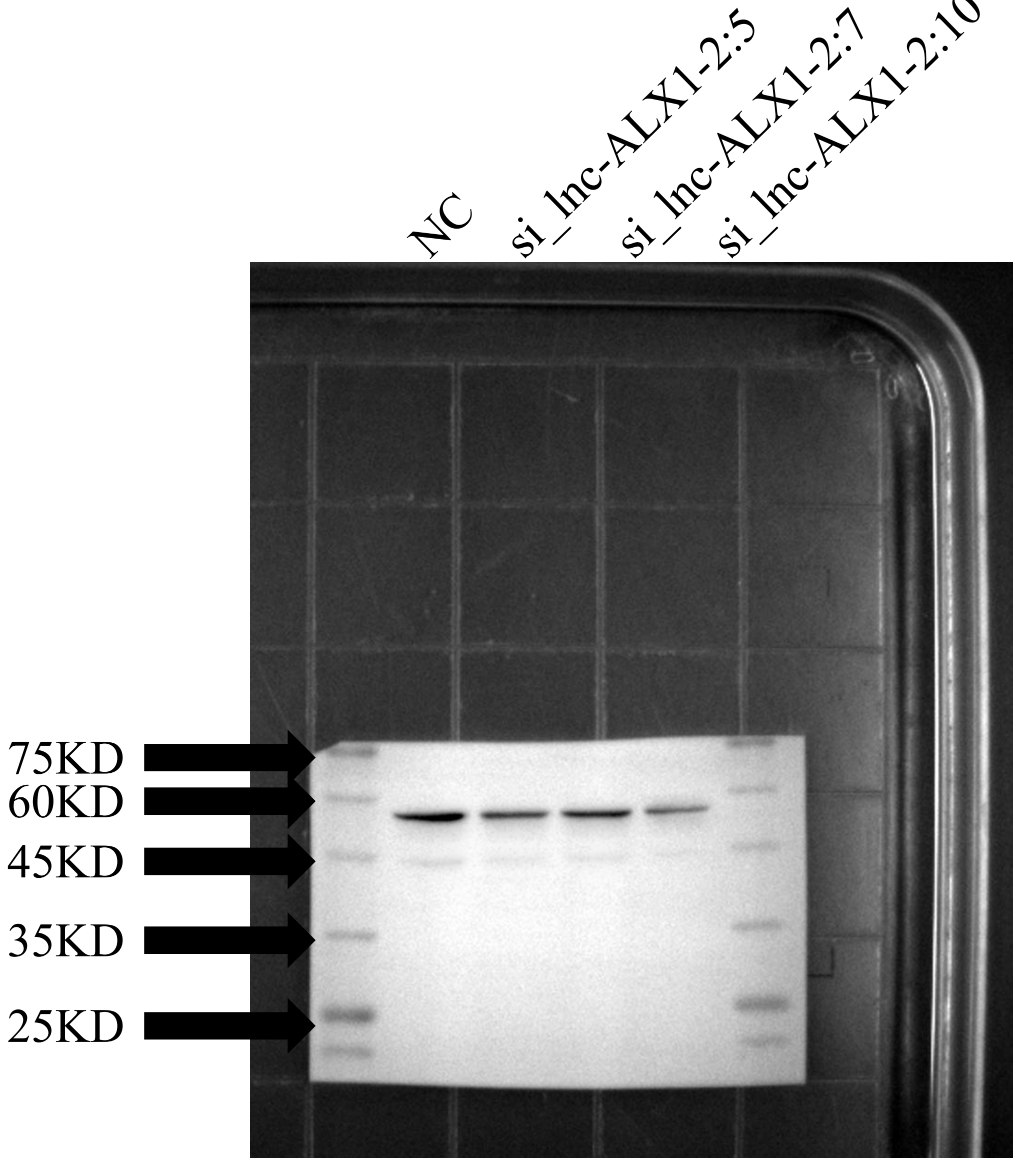

Vimentin (V9), Santa, #sc-6260, 1:500, 43-57KD
HRP-conjugated Affinipure Goat Anti-Mouse IgG(H+L), Proteintech, #SA00001-1; RRID, AB_2722565; 1:1000

Protein Marker (10-245kd), Sangon Biotech, #C620014

Figure 2

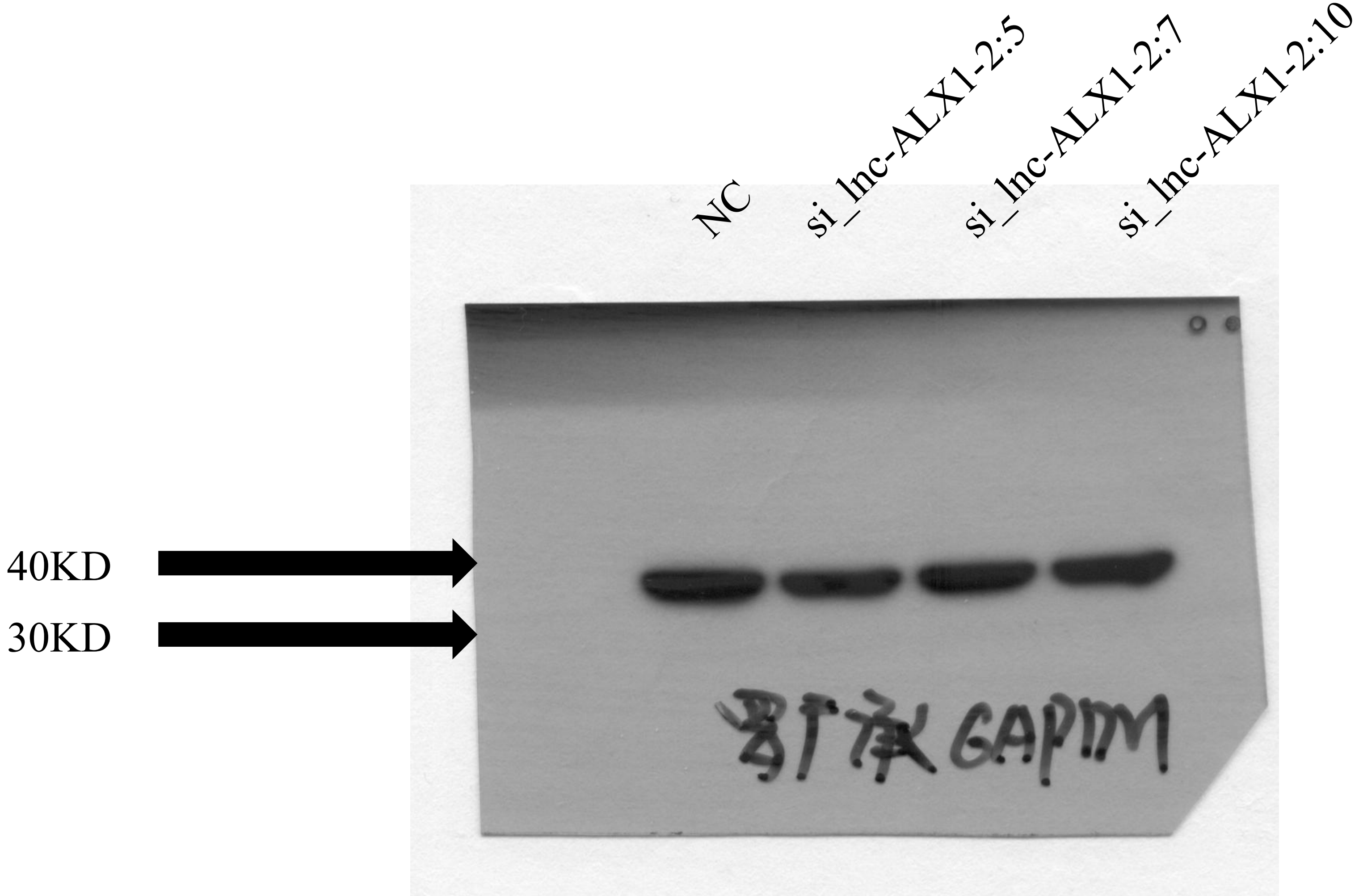

GAPDH Monoclonal antibody, Proteintech, #60004-1-Ig, RRID, AB_2107436; 1:10000, 36KD
HRP-conjugated Affinipure Goat Anti-Mouse IgG(H+L), Proteintech, #SA00001-1; RRID, AB_2722565; 1:5000

Blue Plus II Protein Marker (14-120 kDa), Transgen, #DM111-02

Figure 3

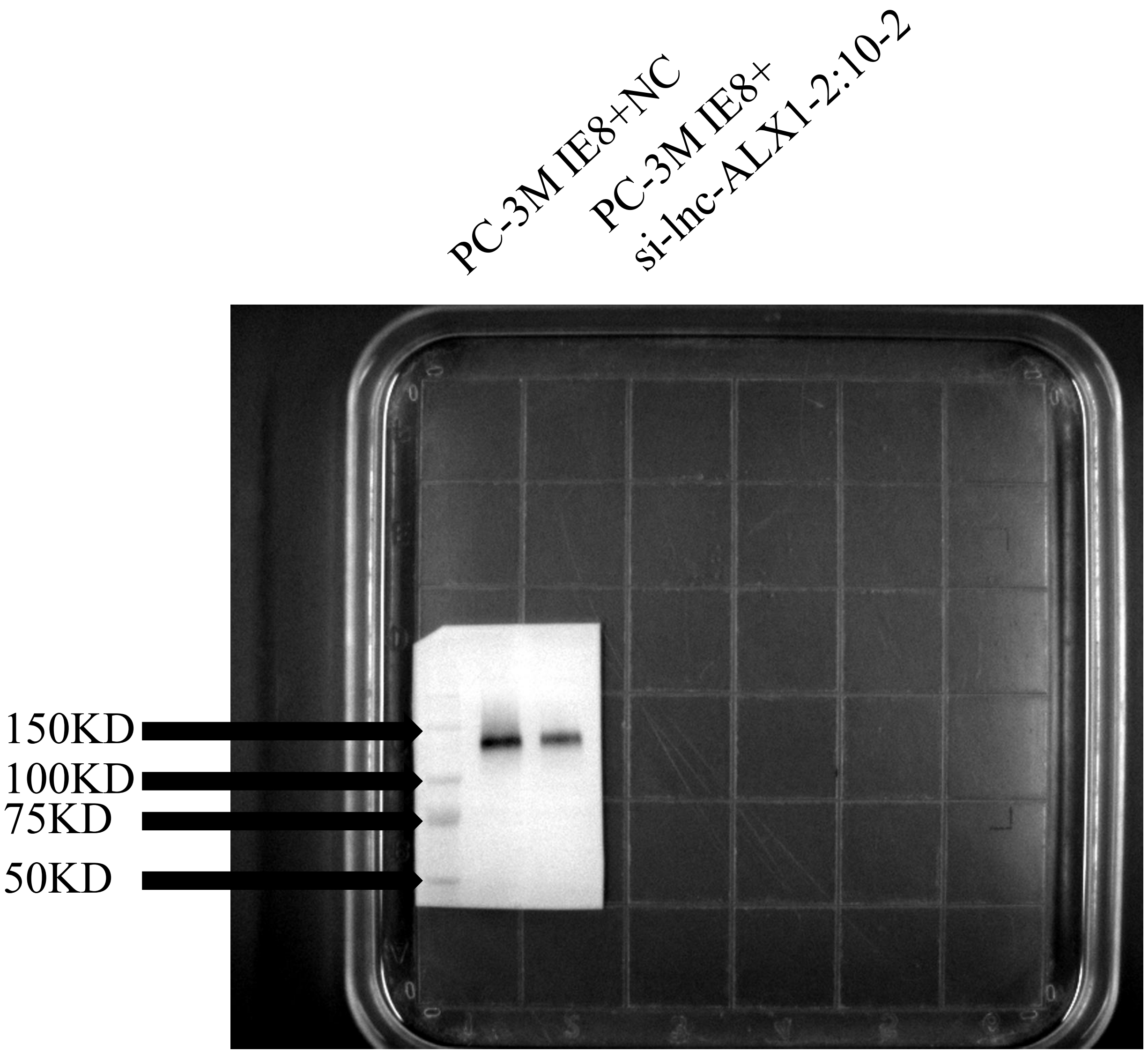

Anti-N Cadherin, santa, sc53488, 1:500, 130KD
HRP-conjugated Affinipure Goat Anti-Mouse IgG(H+L), Proteintech, #SA00001-1; RRID, AB_2722565; 1:1000

Precision Plus Protein™ Dual Xtra Standards, Biorad, #161-0377

Figure 3

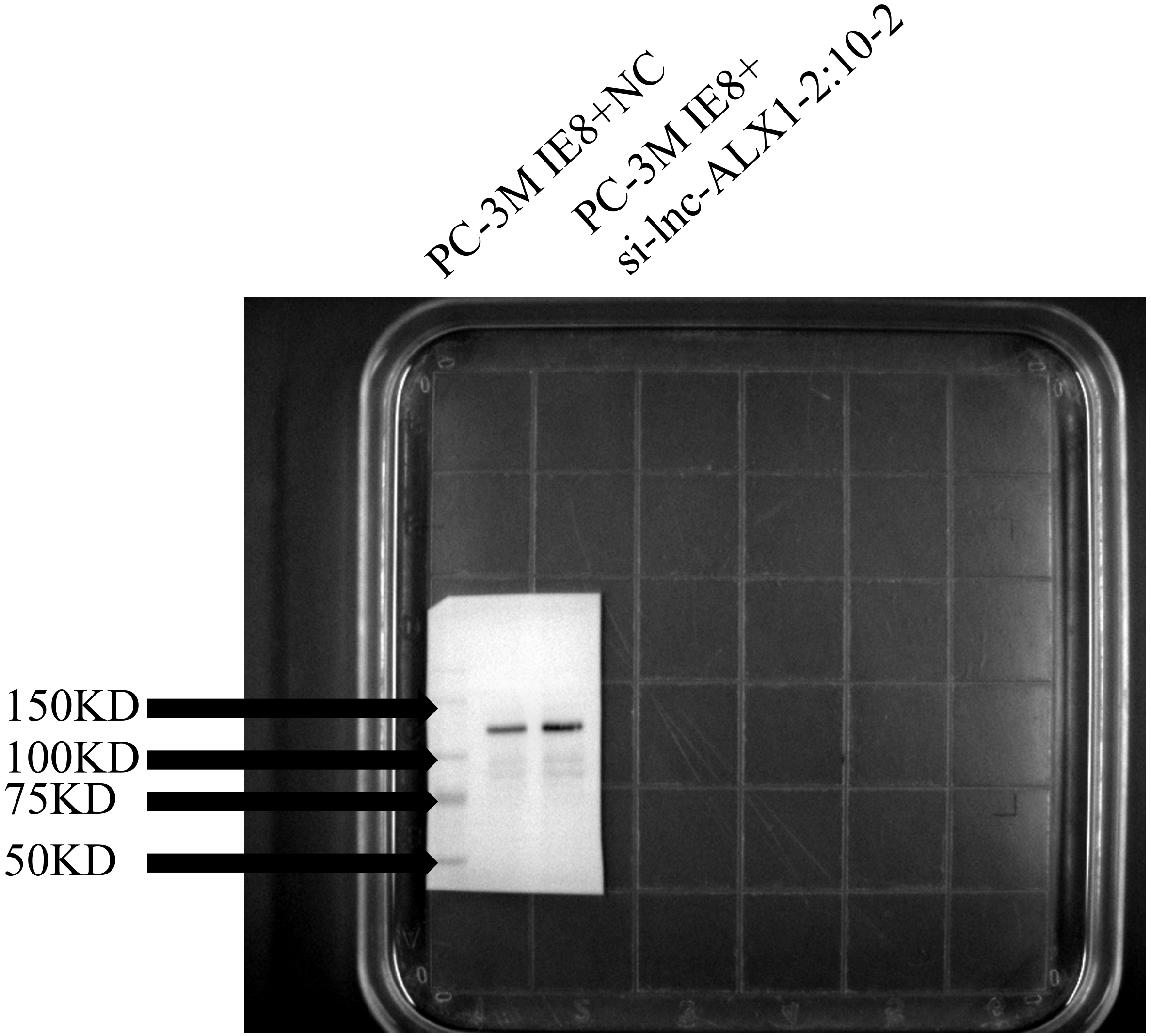

E-cadherin Polyclonal antibody, Proteintech, #20874-1-AP; RRID, AB_10697811; 1:4000, 120-125KD
HRP-conjugated Affinipure Goat Anti-Rabbit IgG(H+L), Proteintech, # SA00001-2; RRID, AB_27225641; 1:5000

Precision Plus Protein™ Dual Xtra Standards, Biorad, #161-0377

Figure 3

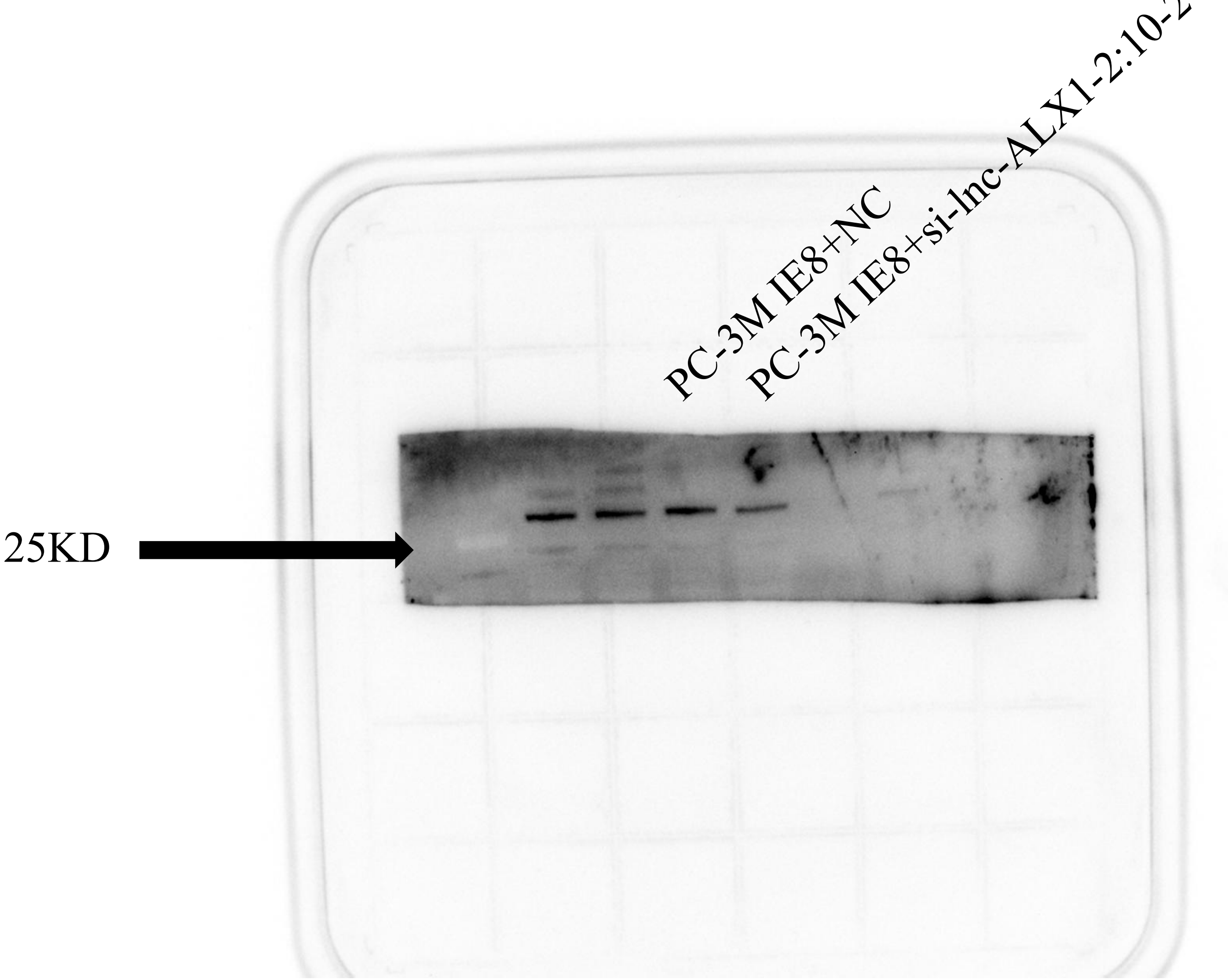

SNAI 1 (G-7), Santa, #sc-271977, 1:1000, 29KD
HRP-conjugated Affinipure Goat Anti-Mouse IgG(H+L), Proteintech, #SA00001-1; RRID, AB_2722565; 1:1000

Precision Plus Protein™ Dual Xtra Standards, Biorad, #161-0377

Figure 3

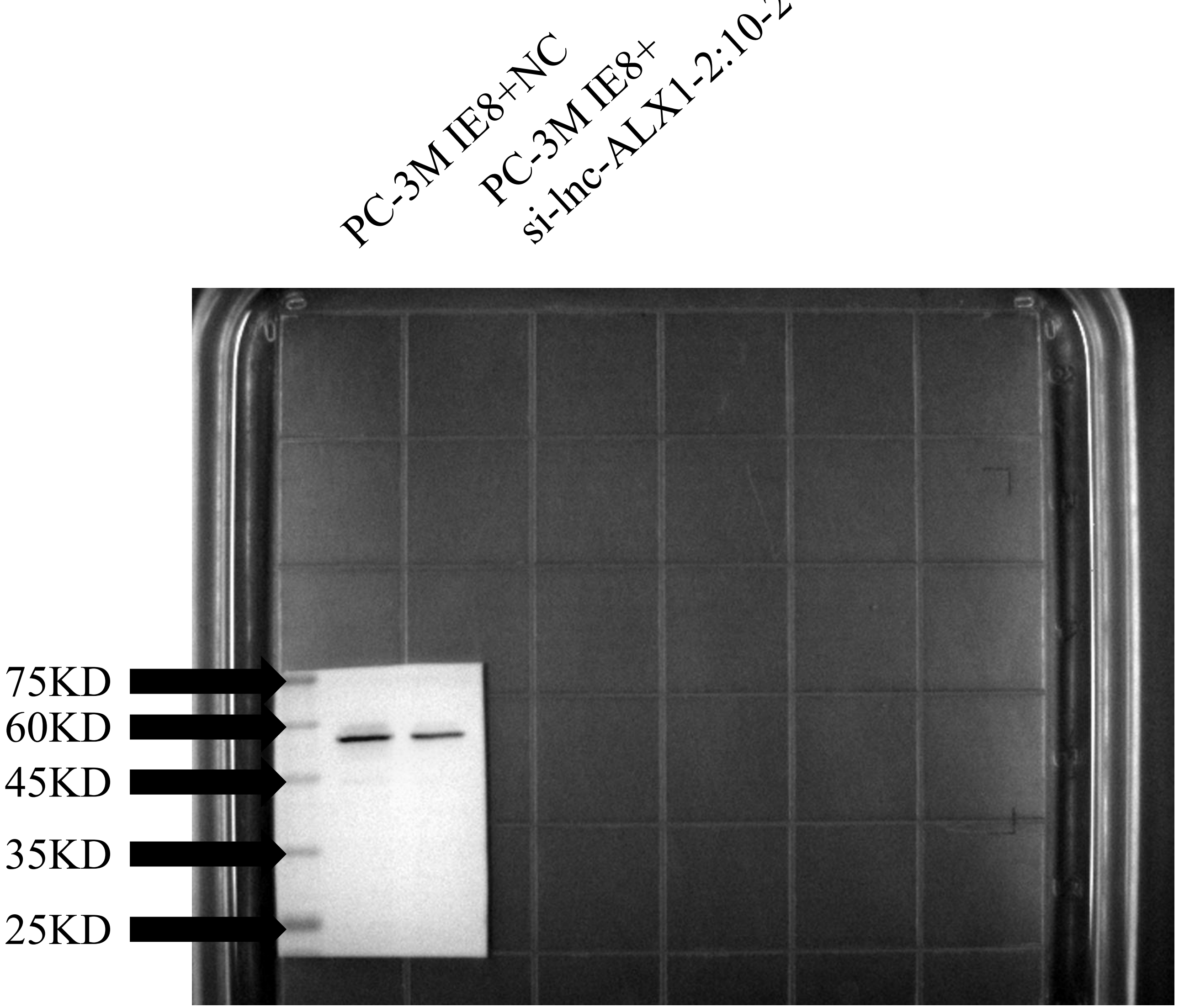

Vimentin (V9), Santa, #sc-6260, 1:500, 43-57KD
HRP-conjugated Affinipure Goat Anti-Mouse IgG(H+L), Proteintech, #SA00001-1; RRID, AB_2722565; 1:1000

Protein Marker (10-245kd), Sangon Biotech, #C620014

Figure 3

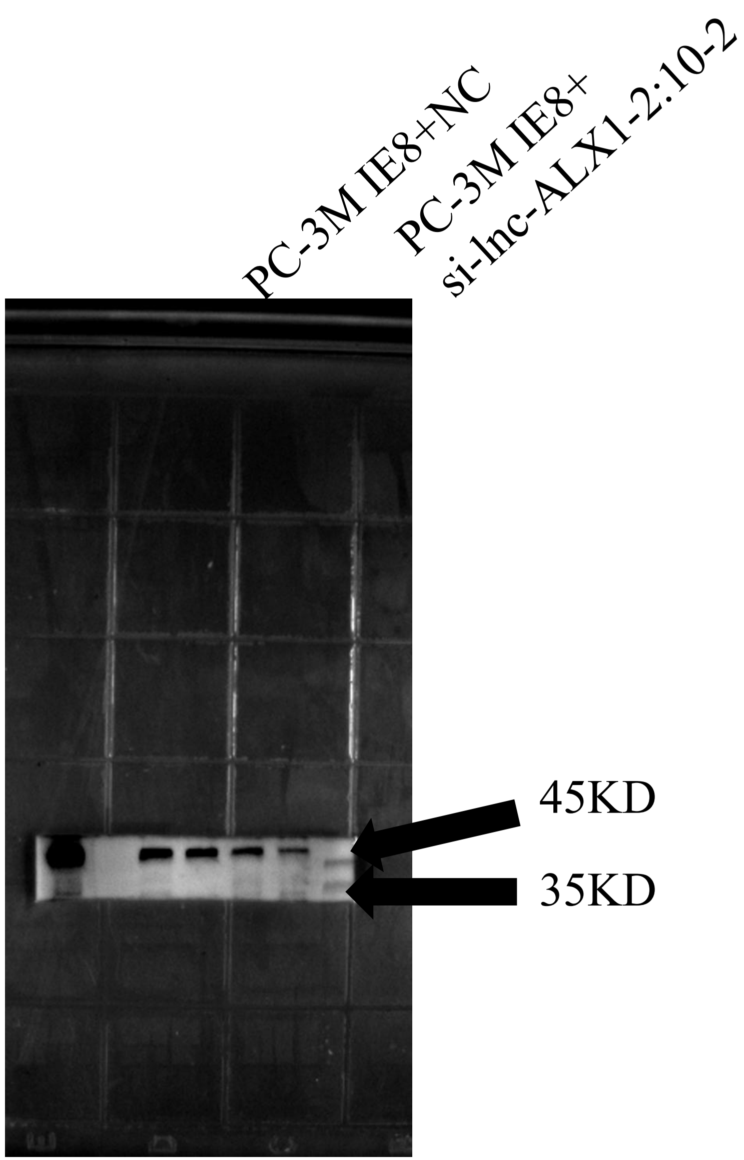

CCNE1, #ab33911, 1:500, 47-50KD
HRP-conjugated Affinipure Goat Anti-Rabbit IgG(H+L), Proteintech, # SA00001-2; RRID, AB_27225641; 1:1000

Protein Marker (10-245kd), Sangon Biotech, #C620014

Figure 3

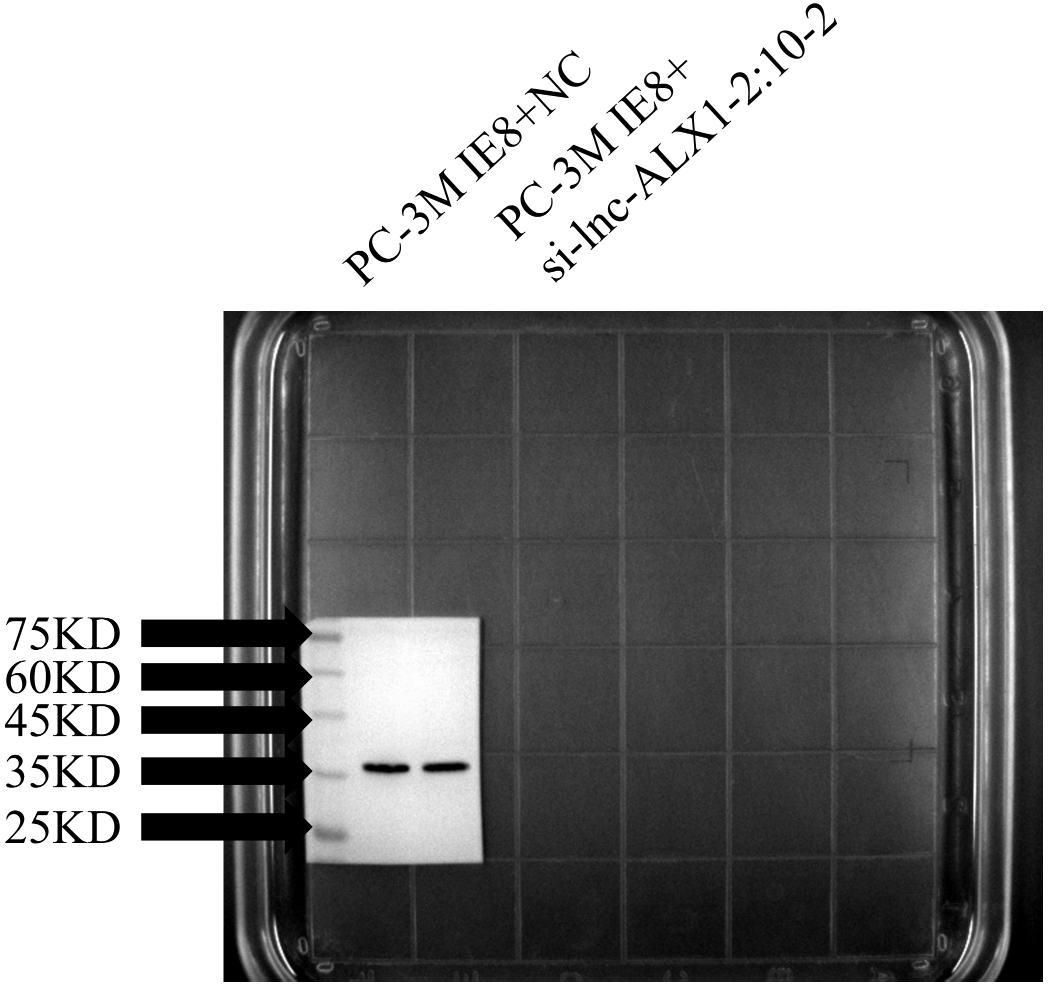

GAPDH Monoclonal antibody, Proteintech, #60004-1-Ig, RRID, AB_2107436; 1:10000, 36KD
HRP-conjugated Affinipure Goat Anti-Mouse IgG(H+L), Proteintech, #SA00001-1; RRID, AB_2722565; 1:5000

Protein Marker (10-245kd), Sangon Biotech, #C620014

Figure 4

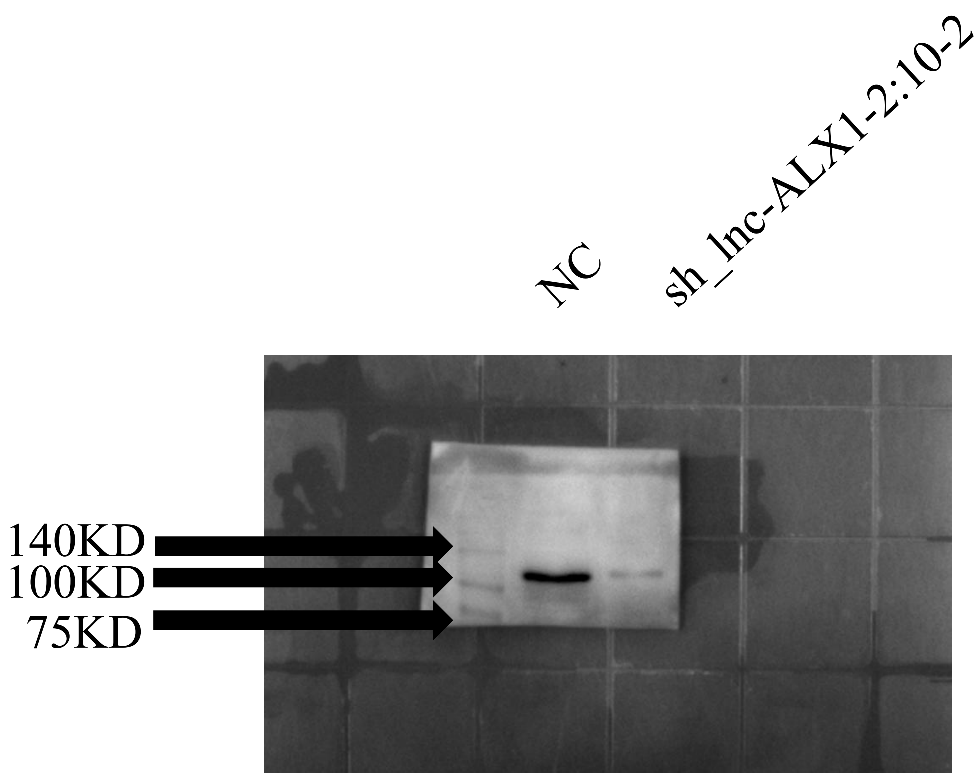

Anti-N Cadherin, santa, sc53488, 1:500, 130KD
HRP-conjugated Affinipure Goat Anti-Mouse IgG(H+L), Proteintech, #SA00001-1; RRID, AB_2722565; 1:1000

Protein Marker (10-245kd), Sangon Biotech, #C620014

Figure 4

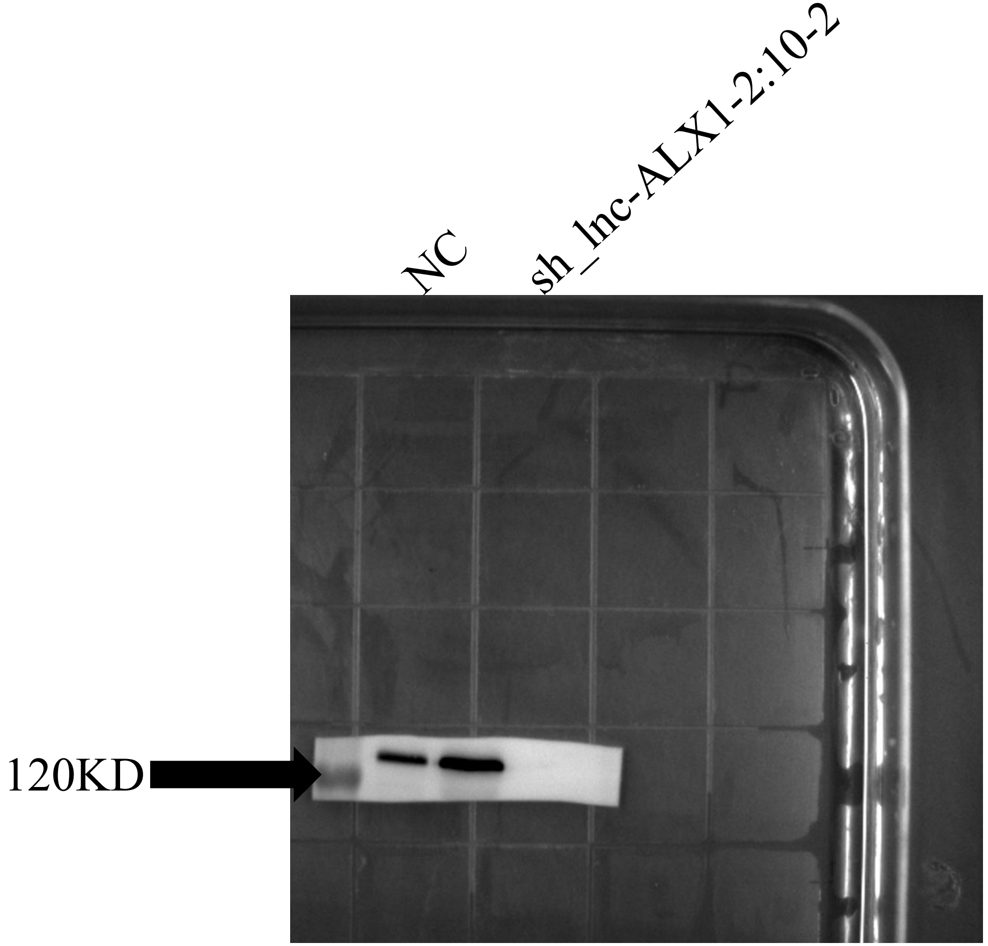

E-cadherin Polyclonal antibody, Proteintech, #20874-1-AP; RRID, AB_10697811; 1:4000, 120-125KD
HRP-conjugated Affinipure Goat Anti-Rabbit IgG(H+L), Proteintech, # SA00001-2; RRID, AB_27225641; 1:5000

Blue Plus II Protein Marker (14-120 kDa), Transgen, #DM111-02

Figure 4

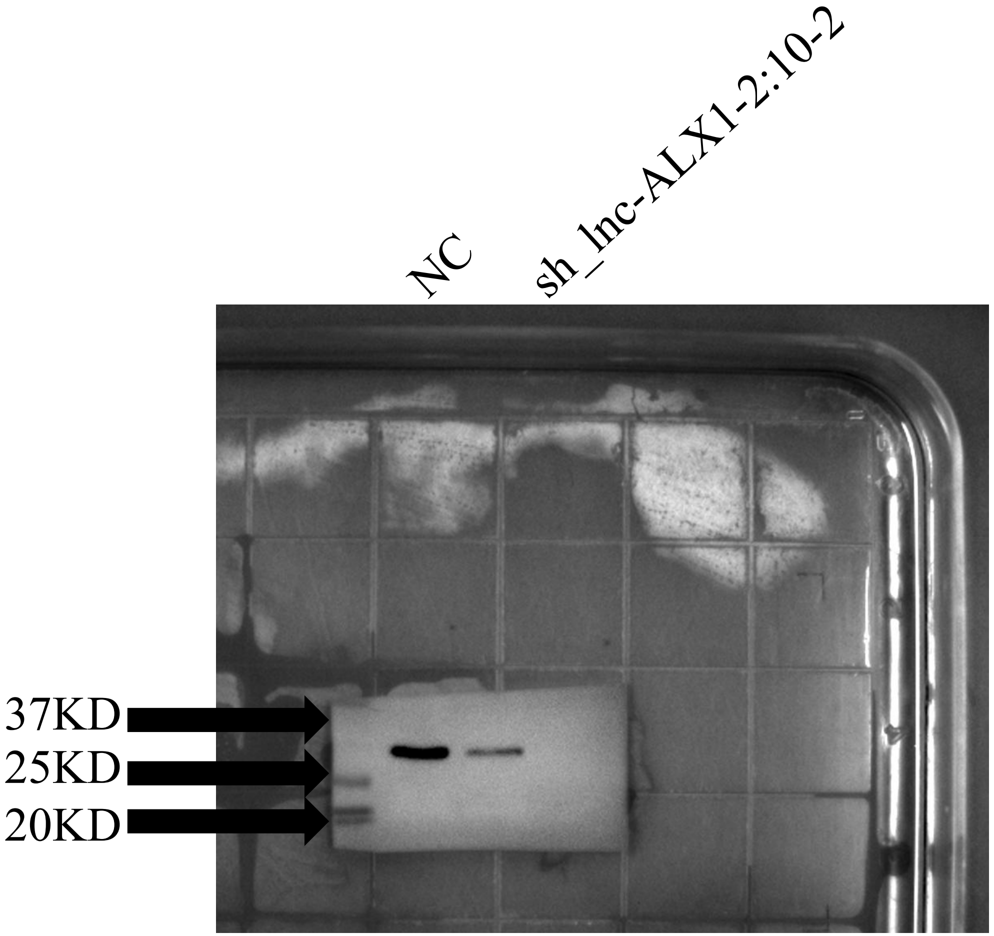

SNAI 1 (G-7), Santa, #sc-271977, 1:1000, 29KD
HRP-conjugated Affinipure Goat Anti-Mouse IgG(H+L), Proteintech, #SA00001-1; RRID, AB_2722565; 1:1000

Precision Plus Protein™ Dual Xtra Standards, Biorad, #161-0374

Figure 4

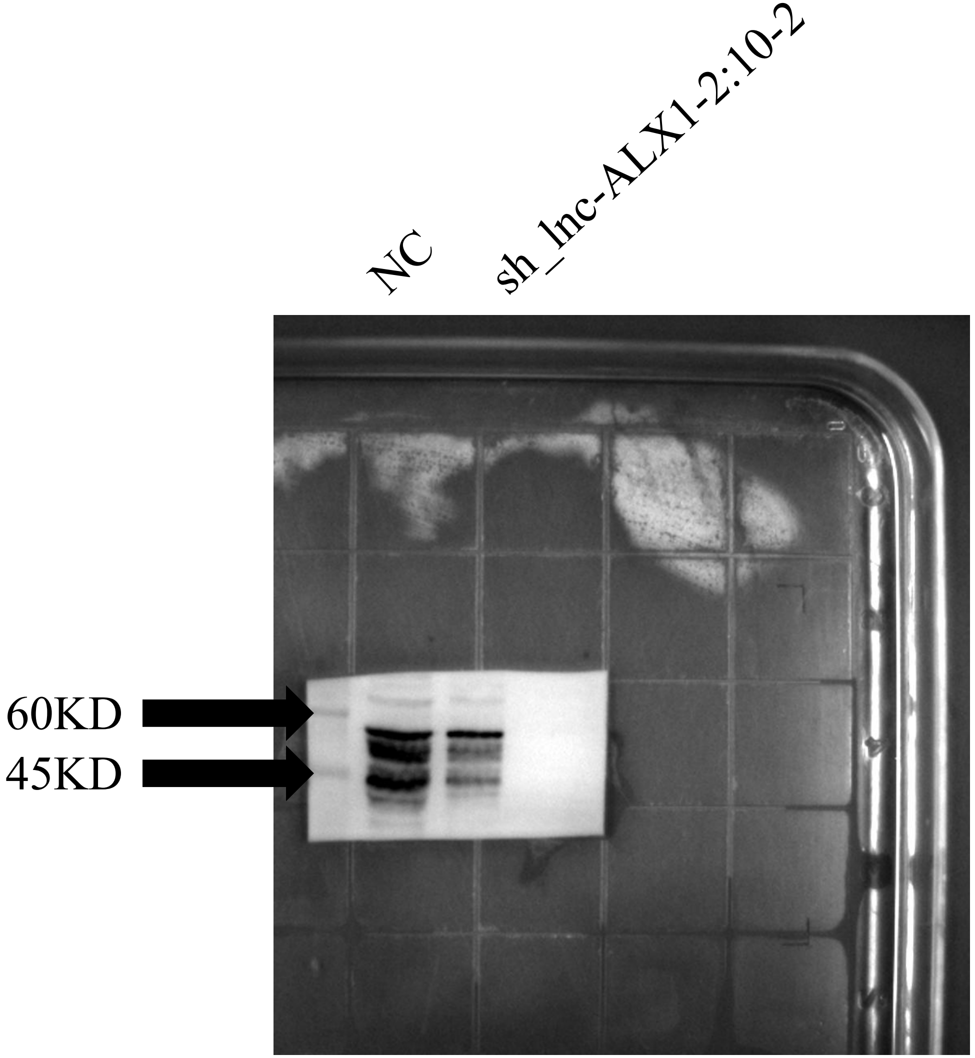

Vimentin (V9), Santa, #sc-6260, 1:500, 43-57KD
HRP-conjugated Affinipure Goat Anti-Mouse IgG(H+L), Proteintech, #SA00001-1; RRID, AB_2722565; 1:1000

Protein Marker (10-245kd), Sangon Biotech, #C620014

Figure 4

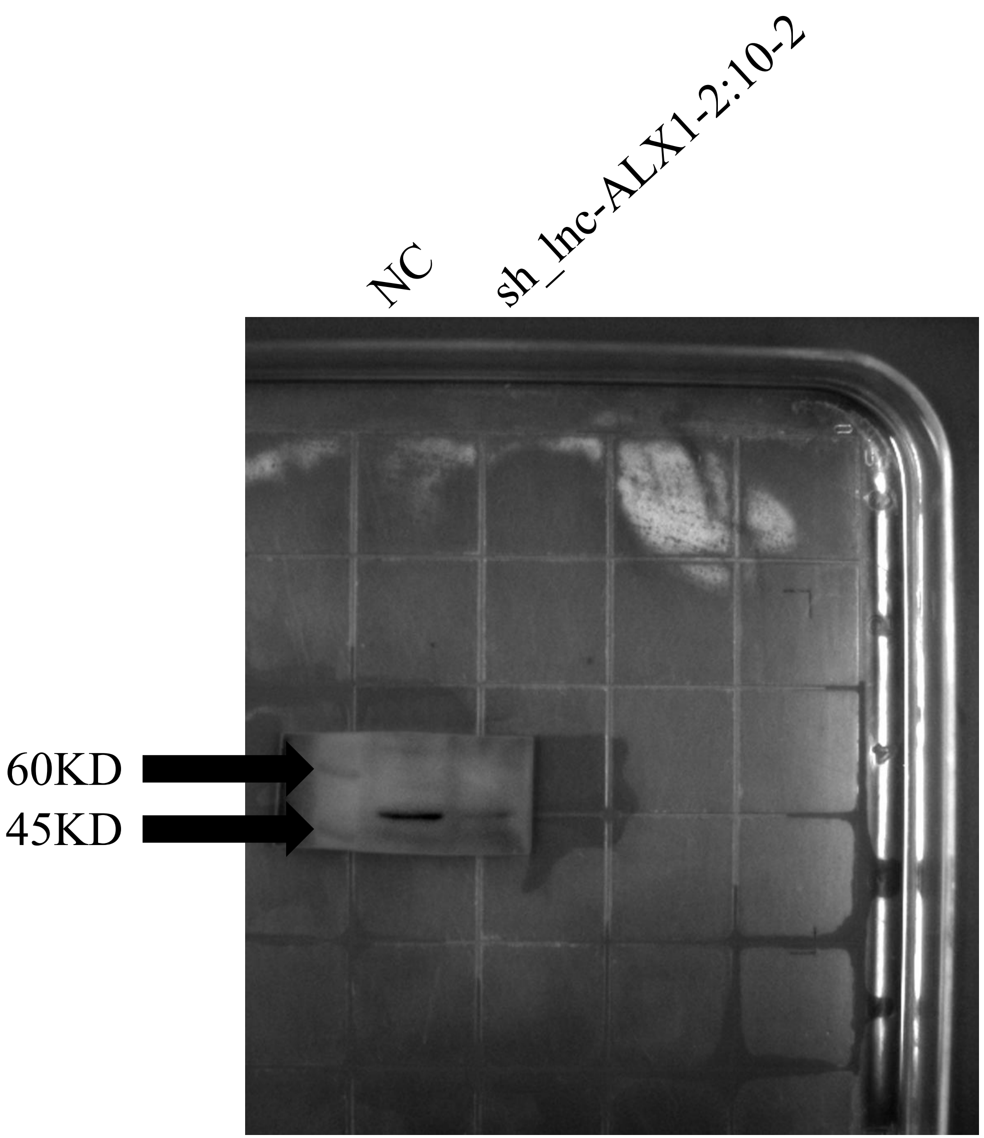

CCNE1, #ab33911, 1:500, 47-50KD
HRP-conjugated Affinipure Goat Anti-Rabbit IgG(H+L), Proteintech, # SA00001-2; RRID, AB_27225641; 1:1000

Protein Marker (10-245kd), Sangon Biotech, #C620014

Figure 4

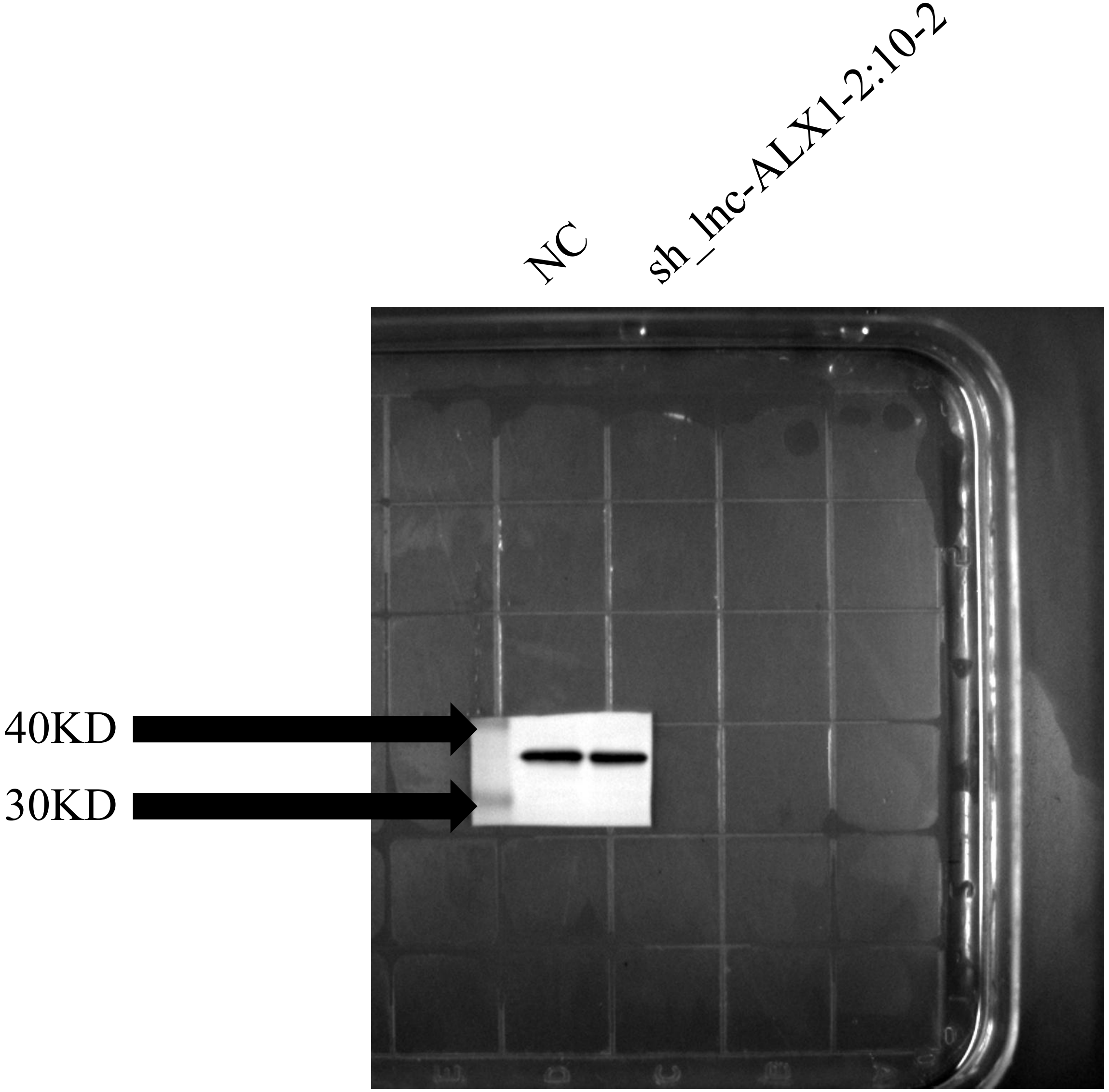

GAPDH Monoclonal antibody, Proteintech, #60004-1-Ig, RRID, AB_2107436; 1:10000, 36KD
HRP-conjugated Affinipure Goat Anti-Mouse IgG(H+L), Proteintech, #SA00001-1; RRID, AB_2722565; 1:5000

Blue Plus II Protein Marker (14-120 kDa), Transgen, #DM111-02
